# ExermiR‐129‐3p Enhances Muscle Function by Improving Mitochondrial Activity Through PARP1 Inhibition

**DOI:** 10.1002/jcsm.13823

**Published:** 2025-04-20

**Authors:** Yeo Jin Shin, Jae Won Yang, Heeyeon Jeong, Joyeong Kim, Bora Lee, Ji‐Won Kim, Seung‐Min Lee, Ju Yeon Kwak, Young Hoon Son, Kap Jung Kim, Yong Ryoul Yang, Chuna Kim, Ki‐Sun Kwon, Kwang‐Pyo Lee

**Affiliations:** ^1^ Aging Convergence Research Center Korea Research Institute of Bioscience and Biotechnology (KRIBB) Daejeon Republic of Korea; ^2^ Department of Bioscience KRIBB School, Korea University of Science and Technology (UST) Daejeon Republic of Korea; ^3^ Department of Bioinformatics KRIBB School, Korea University of Science and Technology (UST) Daejeon Republic of Korea; ^4^ Department of Medical Science Chungnam National University College of Medicine Daejeon Republic of Korea; ^5^ Biohybrid Systems Group, Coulter Department of Biomedical Engineering Georgia Institute of Technology & Emory University School of Medicine Atlanta Georgia USA; ^6^ Department of Orthopedic Surgery Eulji University College of Medicine Daejeon Republic of Korea; ^7^ Aventi Inc. Daejeon Republic of Korea

**Keywords:** exercise mimic, exermiR, microRNA, PARP1, skeletal muscle, TRIM63

## Abstract

**Background:**

Physical exercise has beneficial effects on various organs, including skeletal muscle. However, not all patients are capable of engaging in exercise to maintain muscle function, which underscores the importance of identifying molecular mechanisms of physical training that could lead to the discovery of exercise‐mimicking molecules.

**Methods:**

This study sought to identify molecular mediators of exercise that could improve muscle function. We focused on the exercise‐induced microRNA (miR)‐129‐3p, investigating its role and effects on mitochondrial activity both in vivo and in vitro. The expression of miR‐129‐3p was analysed in skeletal muscle following exercise, and its downstream effects on the poly (ADP‐ribose) polymerase‐1 (Parp1)‐SIRT1‐PGC1α signalling pathway were elucidated. Functional studies were conducted using muscle‐specific overexpression of miR‐129‐3p in adult mice and intramuscular injection of AAV9‐miR‐129‐3p in obese mice to assess exercise capacity and muscle strength.

**Results:**

Exercise was found to upregulate miR‐129‐3p in skeletal muscle (*p* < 0.05), which directly inhibits *Parp1*, a major NAD^+^‐consuming enzyme. This inhibition leads to increased NAD^+^ levels (*p* < 0.05), activating SIRT1 and subsequently reducing the acetylation of PGC1α, thereby enhancing mitochondrial function. Muscle‐specific overexpression of miR‐129‐3p in adult mice significantly enhanced exercise capacity (> 130%, *p* < 0.0001), while AAV9‐miR‐129‐3p injections ameliorated muscle weakness (twitch force, > 140%, *p* < 0.05; tetanic force, > 160%, *p* < 0.01) in obese mice. In human skeletal muscle myoblasts, miR‐129‐3p improved mitochondrial function via the PARP1‐SIRT1‐PGC1α signalling pathway.

**Conclusion:**

Our findings suggest that miR‐129‐3p, induced by exercise, can mimic the beneficial effects of physical exercise. This highlights miR‐129‐3p as a potential therapeutic target for improving muscle health, especially in individuals unable to exercise.

**Abbreviations**
miRNA: microRNA
PARP1: poly (ADP‐ribose) polymerase‐1
NAD: nicotinamide adenine dinucleotide
Myl1: myosin light polypeptide 1
UTR: untranslated region
TA: tibialis anterior
M: mimic
I: inhibitor
CSA: cross section area
Ctrl: control
AAV9: adeno‐associated virus serotype 9
HSMM: human skeletal muscle myoblast

## 1 Introduction

Physical exercise is one of the best methods to keep our bodies healthy, and it is recommended as a nonpharmacological treatment for a wide range of chronic illnesses, including cancer, metabolic disorders, and cardiovascular diseases [1, 2, 3]. However, meeting the recommended levels of physical exercise can be challenging, particularly for patients who are unable to exercise [4]. Therefore, developing therapeutic strategies that can recapitulate the beneficial effects of exercise is an attractive approach for improving muscle function when physical exercise is not feasible. Exercise enhances physical capabilities, but the molecular mechanisms responsible for its beneficial effects on muscle tissues are still not fully understood.

MicroRNAs (miRNAs), small, noncoding RNAs with a length of ~22 nucleotides, have been shown to play critical roles in regulating numerous biological processes, including muscle physiology [5, 6, 7]. MiRNAs are involved in post‐transcriptional regulation of gene expression by degrading mRNAs or by suppressing the translation of their targets [5, 8]. Expression of miRNAs is altered in skeletal muscle after exercise [9, 10], and previous studies have identified several miRNAs that are differentially regulated in skeletal muscle in response to acute [11], endurance [12] or resistance training [13]. Components of the miRNA biogenesis machinery, *Drosha, Dicer* and *Exportin‐5*, are upregulated in skeletal muscle after exercise [11, 13]. These results suggest a potential role for miRNAs in regulating muscle adaptation in response to physical exercise. However, it remains unclear how miRNAs regulate the beneficial effects of exercise and which pathways are the main targets of their activity.

Here, miRNAs regulate numerous biological processes, and we hypothesized that exercise‐induced miRNAs in skeletal muscle (*exermiRs*) play a critical role in improving muscle endurance, potentially serving as therapeutic targets for individuals unable to perform regular physical activity. We performed a comparative analysis of miRNA expression profiles in skeletal muscle after exercise both in vivo and in vitro, using a cellular exercise model, which led to our identification of an exercise‐induced miRNA, miR‐129‐3p. Through further investigation, we demonstrated that miR‐129‐3p regulates multiple target genes, including *Parp1* and *Trim63*, to improve mitochondrial activity as well as muscle atrophy. We then validated the therapeutic efficacy of miR‐129‐3p in both obese and muscle atrophy mouse models. Our results suggest that strategies aimed at upregulating *exermiRs* could represent a valuable approach to mimic exercise benefits.

## 2 Methods

### 2.1 Animal Studies

WT (6‐month‐old) C57BL/6 mice were purchased from Damual Science (Daejeon, Korea). Before starting the exercise training, a 1‐week adaptation period was implemented to familiarize the mice with the treadmill (Daehan Biolink, Korea). After this adaptation period, the mice underwent a progressive exercise training regimen for 5 days per week. The training started with running at 12 m/min for 30 min in the first week, with the speed increasing by 2 m/min each subsequent week, reaching 18 m/min by the fourth week. Muscle tissues were collected immediately after the final exercise session from both the control and exercised mice and subsequently analysed. To measure running capacity, mice were tested on a treadmill starting at 12 m/min, with the speed increasing by 2 m/min every 10 min until reaching 20 m/min. To overexpress miRNA in whole‐muscle tissues, 100 μL (1 × 10^12^ GC) of AAV9‐lsl‐GFP‐miR‐129‐3p, AAV9 encoding the loxp‐stop‐loxp‐GFP‐miR‐129‐3p cassette (Applied Biological Materials Inc., Canada), was injected into the tail vein of WT or Myl1‐Cre mice using a 26G 1/2″ needle connected to a syringe. Myl1‐Cre mouse was purchased from the Jackson Laboratory (US). The mice treated with AAV9 were euthanized 4‐weeks postinjection, and the muscle tissues that were separated were utilized for additional examination. For intramuscular injection, 50 μL (1 × 10^10^ GC) of AAV9‐Ctrl or AAV9‐miR‐129‐3p (Applied Biological Materials Inc., Canada) was directly injected into TA muscle or contralateral TA muscle of either adult or *ob/ob* mice using a 29G needle connected to an insulin syringe. The *ob/ob* mice were purchased from the SLC (Shizuoka, Japan). All mice in this study were fed a standard laboratory diet (3.1 kcal/g). Experiments with mice and viruses were carried out in accordance with established methods approved by the Animal Care and Use Committee of KRIBB.

### 2.2 Additional Methods

Additional details about the materials and methods are provided in the Supporting Information section.

### 2.3 Statistical Analysis

All data are shown as means ± SEM, unless otherwise specified. All experimental data, including luciferase activity, qPCR and Western blot data, were normalized to the appropriate control (Renilla luciferase, housekeeping genes or proteins) and compared to the respective control condition, such as in vitro control cells, in vivo wild‐type or contralateral muscles. Data were analysed by using GraphPad Prism (GraphPad Software Inc., USA). Comparisons between two groups were conducted with Student's *t*‐test or Mann–Whitney test. For comparisons involving more than two groups, one‐way analysis of variance (ANOVA) with Turkey's post hoc test was used to determine statistical significance. For multiple comparisons involving different groups and outcomes, a parametric two‐way ANOVA was used. *p*‐values less than 0.05 were regarded as statistically significant.

## 3 Results

### 3.1 miR‐129‐3p Is Upregulated in Skeletal Muscle After Physical Exercise Training

**FIGURE 1:**
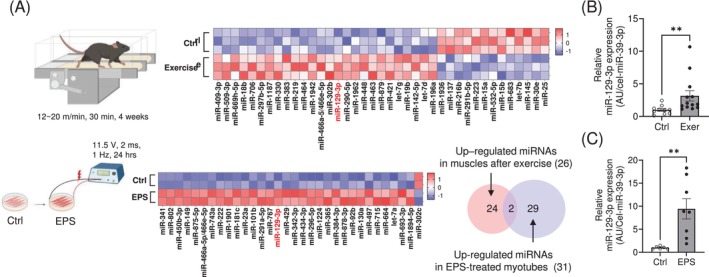
miR‐129‐3p is increased in skeletal muscle following exercise training. MiRNA profiling in Ctrl vs. exercised mouse muscle and Ctrl vs. EPS‐treated C2C12 myotubes. (A) The heatmaps show 26 upregulated miRNAs in exercised mouse muscle and the 31 up‐regulated miRNAs in EPS‐treated C2C12 myotubes. Z‐scores are shown. *p* < 0.05. miR‐129‐3p is highlighted in red as the only miRNA conserved in humans that is expressed in both datasets. (B) qRT‐PCR analysis of miR‐129‐3p in exercised mouse muscle (Ctrl, *n* = 10; Exer, *n* = 13) and (C) EPS‐treated C2C12 myotubes (Ctrl, *n* = 6; EPS, *n* = 8). **p < 0.01. Statistical significance was assessed by Mann–Whitney test (for B) or Student's *t*‐test (for C).

To investigate differential expression of miRNAs in the skeletal muscles of mice exposed to chronic exercise, we analysed the miRNA expression profile in muscle tissues isolated from mice and compared them with untrained mice. We found that 26 miRNAs were significantly upregulated in the muscles of trained mice with enhanced exercise capacity (Figure S1A and Table S1). To focus on myocyte‐specific miRNA upregulation using an orthogonal approach, we employed an in vitro exercise model recently developed in our laboratory in which electrical pulse stimulation (EPS) is applied to cultivated C2C12 myotubes [14]. Comparative analysis of miRNA expression profiles in muscles and in EPS‐treated myotubes (Table S2) uncovered two miRNAs, miR‐129‐3p and miR‐466a‐5p/466e‐5p (Figure 1A). We selected the human‐conserved miR‐129‐3p and validated its expression in muscle tissues after exercise and in EPS‐treated myotubes (Figure 1B,C).

### 3.2 Exercise‐Induced miR‐129‐3p Inhibits *Parp1* and *Trim63* Expression by Direct Interaction

**FIGURE 2:**
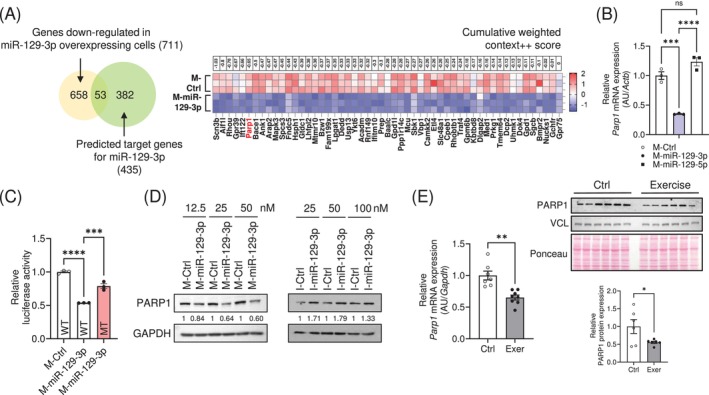
Exercise‐induced miR‐129‐3p modulates PARP1 expression. (A) Comprehensive RNA sequencing analysis identified 711 genes downregulated in C2C12 cells overexpressing miR‐129‐3p, and 435 genes predicted as targets of miR‐129‐3p using the TargetScan algorithm. These genes are sorted by their low cumulative weighted context++ scores. (B) qRT‐PCR analysis of *Parp1* in C2C12 myotubes transfected with the indicated mimics. The data were normalized to *Actb*. (C) Firefly luciferase reporter analysis using WT *Parp1* 3′ UTR or mutant *Parp1* 3′ UTR with deletion of the seed sequence. (D) PARP1 protein abundance in C2C12 myotubes transfected with miR‐129‐3p (*left*) mimics or (*right*) inhibitors. The PARP1 protein levels were normalized using GAPDH and quantified using ImageJ software. (E) qRT‐PCR and immunoblot analysis of *Parp1* expression in the muscle of exercised mice (*n* = 6). The *Parp1* mRNA levels were normalized to *Gapdh*, and the PARP1 protein abundance was normalized to VCL and quantified using ImageJ software. The data are presented as the mean ± SEM. *p < 0.05, **p < 0.01, ***p < 0.001, ****p < 0.0001. Statistical significance was assessed by Student's *t*‐test (for E) or one‐way ANOVA (for B and C).

To identify the target genes of miR‐129‐3p, we performed RNA sequencing analysis in the C2C12 myotubes transfected with a mimic (M‐) of miR‐129‐3p, and found 711 genes significantly downregulated (Figure S1B and Table S3). Using the TargetScan algorithm (www.targetscan.org), we found that 53 genes among them might be putative targets of miR‐129‐3p (Figure 2A and Table S4). Since previous reports revealed that poly (ADP‐ribose) polymerase‐1 (PARP1) could regulate mitochondrial metabolism [15, 16], we primarily focus on *Parp1* among the putative targets. We observed a downregulation of *Parp1* expression in myotubes transfected with M‐miR‐129‐3p (Figure 2B). Reporter analysis using a construct that expresses luciferase‐*Parp1* 3′ UTR and M‐miR‐129‐3p indicated that miR‐129‐3p reduced luciferase activity. The inhibition was specific, as deletion of the human conserved binding site of miR‐129‐3p diminished this repression (Figure 2C). PARP1 expression was downregulated in myotubes overexpressing M‐miR‐129‐3p and upregulated in those transfected with an inhibitor (I) of miR‐129‐3p (I‐miR‐129‐3p; Figure 2D). In addition, mRNA and protein abundance of PARP1 were robustly downregulated in exercised mouse muscle as expected (Figure 2E). Consistently, *Parp1* mRNA levels were downregulated in electrically stimulated myotubes (Figure S1C).

Since miR‐129‐3p overexpression induced a hypertrophic phenotype while inhibition showed an atrophic phenotype in myotubes (Figure S1D), we examined the expression levels of muscle‐specific E3 ligases, such as *Atrogin‐1* and *Trim63*, that have been well‐known to induce muscle atrophy [17], in our RNA‐seq data. Expression of *Trim63* was significantly downregulated upon treatment with M‐miR‐129‐3p (Figure S1E). Using scanMIRApp (https://ethz‐ins.org/scanMiR/), we identified a human‐conserved miR‐129‐3p binding site on exon 7 of *Trim63*. Reporter analysis using a luciferase‐*Trim63* exon 7 construct and M‐miR‐129‐3p demonstrated that miR‐129‐3p reduced luciferase activity. This inhibition was reversed when the miR‐129‐3p binding site was deleted (Figure S1F). In addition to computationally identifying miR‐129‐3p targets, we performed pathway enrichment analyses on differentially expressed genes (DEGs) from RNA‐seq data to characterize its transcriptomic effects. The analysis revealed that miR‐129‐3p regulates multiple biological processes, including lipid homeostasis, actin filament organization, myoblast differentiation, mitochondrial organization and neuronal regulation (Figure S1G). Collectively, these results demonstrate that exercise‐induced miR‐129‐3p orchestrates diverse biological processes in skeletal muscle by modulating numerous genes, including its direct targets *Parp1* and *Trim63*.

### 3.3 miR‐129‐3p Enhances Mitochondrial Activity Through the PARP1‐SIRT1‐PGC1α Signalling Pathway

**FIGURE 3:**
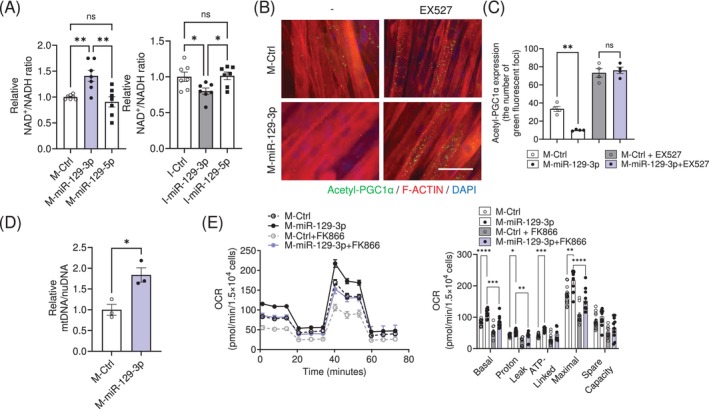
miR‐129‐3p promotes mitochondrial function through the PARP1‐SIRT1‐PGC1α axis. C2C12 myotubes were transfected with M‐miR‐129‐3p (*n* = 7) or I‐miR‐129‐3p (*n* = 7). (A) Intracellular NAD^+^/NADH ratio. (B) Representative images showing decreased acetylation of PGC1α from the Proximity Ligation Assay in C2C12 myotubes transfected with M‐129‐3p and treatment with EX527. *Green*, Acetyl‐PGC1α; *Red*, F‐actin; *Blue*, 4′,6‐diamidino‐2‐phenylindole (DAPI). Scale bar, 100 μm. (C) Quantification of green fluorescent foci (*n* = 4). (D) qRT‐PCR analysis of *MT‐CO1*/*Lpl* ratio to measure mitochondrial DNA content. (E) Oxygen consumption rates (OCR) in C2C12 myotubes transfected with the indicated mimics. FK866, a NAMPT inhibitor. The data are presented as the mean ± SEM. *p < 0.05, **p < 0.01, ***p < 0.001, ****p < 0.0001. Statistical significance was assessed by Student's *t*‐test (for D), one‐way ANOVA (for A) or two‐way ANOVA (for C and E).

PARP1 is a major NAD^+^‐consuming enzyme [18, 19]. *Parp1* knockout mice exhibited activation of SIRT1, an NAD^+^‐dependent deacetylase, due to the accumulation of NAD^+^ contents in skeletal muscle [16]. Of note, NAD^+^ content was significantly accumulated in myotubes transfected with M‐miR‐129‐3p, and NAD^+^ levels were reduced upon I‐miR‐129‐3p transfection (Figure 3A). SIRT1 was activated in M‐miR‐129‐3p overexpressing myotubes by Proximity Ligation Assay (Figure 3B). This reduction was abolished by treatment with EX527, a SIRT1 inhibitor [20] (Figure 3C). We further confirmed that SIRT1 was activated upon M‐miR‐129‐3p transfection, since the acetylation levels of p53, one of the targets for SIRT1, were reduced (Figure S2A). Mitochondrial DNA content was significantly higher (Figure 3D). Consistent with the measures of mitochondrial content, oxygen consumption rates (OCR) were enhanced in those cells. These events were diminished by treatment with FK866, a NAMPT inhibitor that reduces NAD^+^ levels [21] (Figure 3E), or SR18292, a PGC1α inhibitor [20, 22] (Figure S2B). The OCR data was normalized to cell number since miR‐129‐3p did not affect cellular proliferation (Figure S2C). These results indicated that miR‐129‐3p could activate mitochondrial respiration through the PARP1‐SIRT1‐PGC1α axis.

### 3.4 Muscle‐Specific Overexpression of miR‐129‐3p Increases Exercise Capacity in Adult Mice

**FIGURE 4:**
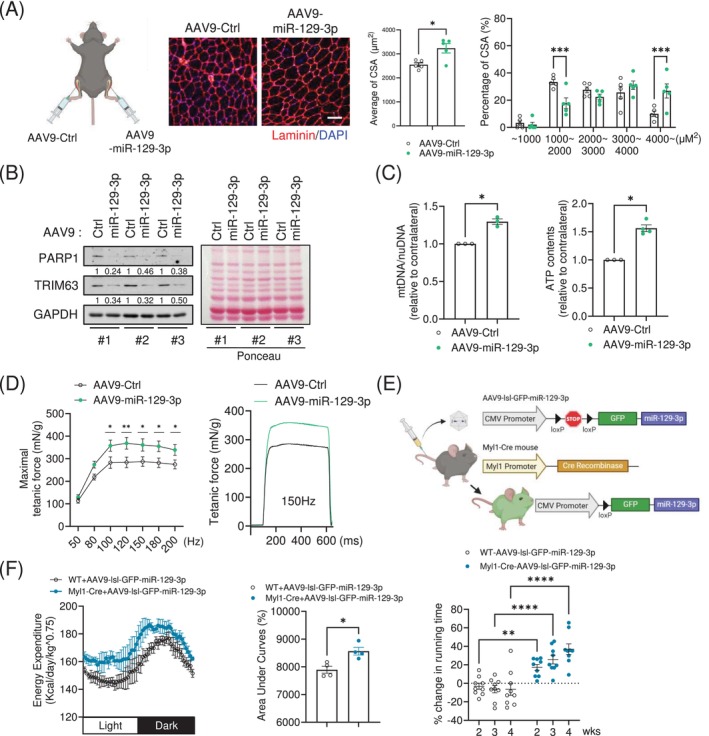
miR‐129‐3p enhances physical activity in adult mice. (A) (*Left*) Diagram of AAV9‐miR‐129‐3p intramuscular injections to overexpress miR‐129‐3p in the tibialis anterior (TA) muscle of 6‐month‐old C57BL6 mice. (*Center*) Representative images of muscle sections immunostained with Laminin (*red*) and DAPI (*blue*). Scale bar, 50 μm. (*Right*) quantification of the average myofiber CSA and its distribution. (B) PARP1 and TRIM63 protein abundance (*n* = 3). Protein expression was normalized using GAPDH. (C) qRT‐PCR analysis of *MT‐CO1*/*Lpl* ratio to measure mitochondrial DNA content. ATP content was assessed in the muscles. *n* = 3. (D) Isometric force (mN/g) measurements using electrical stimulation at frequencies ranging from 50 to 200 Hz and 100 V (*n* = 5). All force measurements were normalized to muscle weight (g). (E) Diagram of AAV9‐lsl‐GFP‐miR‐129‐3p tail vein injections to Myl1‐Cre mice. (F) Energy expenditure was measured over 24 h (*n* = 4). miR‐129‐3p led to an increase in running time during treadmill tests (*n* = 9). The data are presented as the mean ± SEM. *p < 0.05, **p < 0.01, ***p < 0.001, ****p < 0.0001. Statistical significance was assessed by Student's *t*‐test (for A [*left*], C and F [*mid*]) or two‐way ANOVA (for A [*right*], D and F [*right*]).

To investigate the role of miR‐129‐3p in vivo, we performed intramuscular injection of adeno‐associated virus serotype 9 (AAV9) into the tibialis anterior (TA) muscle of 6‐month‐old mice. Notably, TA muscles injected with AAV9‐miR‐129‐3p displayed a significantly larger fibre size compared to the contralateral TA muscles injected with AAV9‐miR‐Ctrl (Figure 4A), even though there were no significant differences in overall muscle weight between the two groups (Figure S3A). Furthermore, the AAV9‐miR‐129‐3p‐injected muscles exhibited lower protein levels of PARP1 and TRIM63 compared to their contralateral counterparts (Figure 4B). Consistent with our in vitro data, acetylation of p53 was reduced after intramuscular injection of AAV9‐miR‐129‐3p (Figure S3B). Muscles injected with AAV9‐miR‐129‐3p exhibited greater mitochondrial DNA content. In addition, we observed higher ATP levels in miR‐129‐3p‐overexpressing muscles (Figure 4C). To investigate whether enlarged TA muscles overexpressing miR‐129‐3p showed improved muscle forces, we measured isometric forces ex vivo using the isolated TA muscle tissues. Although the twitch force of miR‐129‐3p‐overexpressing muscles was not changed (Figure S3C), the maximal tetanic force was markedly higher compared with the contralateral muscles (Figure 4D).

To analyse exercise performance and energy expenditure in vivo, we intravenously administered an AAV9 encoding the loxp‐stop‐loxp‐GFP‐miR‐129‐3p cassette into transgenic mice expressing Cre recombinase under the control of myosin light polypeptide 1 promoter (Myl1‐Cre) for whole muscle‐specific overexpression of miR‐129‐3p (Figure 4E). We validated the overexpression of miR‐129‐3p and GFP in the quadriceps muscles isolated from the hindlimbs of the mice (Figure S3D). Indirect calorimetry analysis using the Comprehensive Lab Animal Monitoring System (CLAMS) revealed that transgenic mice overexpressing miR‐129‐3p in the muscle displayed enhanced energy expenditure without significant changes in respiratory exchange ratio (Figures 4F and S3E). Furthermore, transgenic mice exhibited a greater endurance exercise capacity for 4 weeks after delivery of AAV9 without significant changes in their body weight or grip strength (Figures 4F, S3F and S3G). These results suggest that miR‐129‐3p could act as an exercise mimetic for the enhancement of physical performance in adult lean mice.

### 3.5 Intramuscular Injection of miR‐129‐3p Ameliorates Muscular Weakness in Obese Mice

**FIGURE 5:**
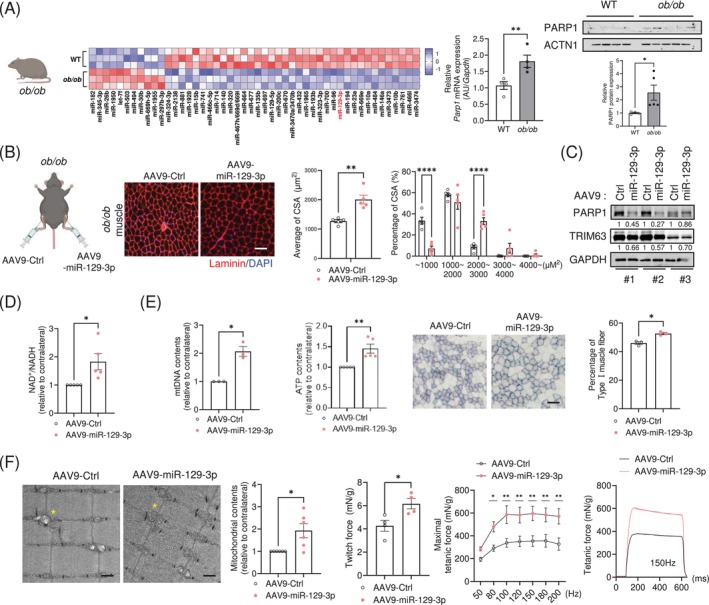
miR‐129‐3p ameliorates muscle weakness in obese mice. MiRNA profiling in WT vs. *ob/ob* mouse muscle. (A) The heatmap shows the 11 upregulated miRNAs and the 37 downregulated miRNAs in *ob/ob* mouse muscle. qRT‐PCR (*n* = 4) and immunoblot (WT; *n* = 4, *ob/ob*; *n* = 5) analysis of the *Parp1* expression. (B) (*Left*) Diagram of AAV9‐miR‐129‐3p intramuscular injections in *ob/ob* mice. (*Right*) Representative images of muscle sections immunostained with Laminin (*red*) and DAPI (*blue*). Quantification of the average myofiber CSA and its distribution. Scale bar, 50 μm. (C) Protein levels of PARP1 and TRIM63 (*n* = 3). Protein expression was normalized to GAPDH levels. (D) Relative NAD^+^/NADH ratio compared to contralateral muscle (*n* = 5). (E) Relative mtDNA contents (*n* = 3). Relative ATP contents (*n* = 5). Representative images of succinate dehydrogenase (SDH) staining and quantification of the percentage of actual Type I fibre (*n* = 3). Scale bar, 50 μm. (F) Representative TEM images. Relative mitochondrial content was measured by numbering the mitochondria per view of images (*n* = 6). The yellow asterisks indicate mitochondria. Scale bars, 500 nm. Twitch and tetanic force measurements (*n* = 4). Twitch forces (1 Hz and 100 V) and tetanic forces (50–200 Hz and 100 V) were determined using an electrical stimulator. All force measurements were normalized to muscle weight (g). The data are presented as the mean ± SEM. *p < 0.05, **p < 0.01, ****p < 0.0001. Statistical significance was assessed by Student's *t*‐test (for A, B [*left*], D, E and F [*left*]) or two‐way ANOVA (for B [*right*] and F [*right*]).

Obesity leads to a substantial decline in the quality of skeletal muscle, which limits effective and continuous physical exercise [23, 24]. MiRNA profiling analysis revealed that miR‐129‐3p was significantly downregulated in muscles isolated from *ob/ob* mice, a hyperphagic mouse model that develops obesity [25, 26], compared to wild‐type mice (Figure 5A and Table S5). In line with our previous observations, lower expression of miR‐129‐3p was associated with higher mRNA and protein levels of PARP1 in the muscles of obese mice. Next, we investigated whether miR‐129‐3p overexpression could improve the muscular weakness exhibited by obese mice. TA muscles injected with AAV9‐miR‐129‐3p had significantly enlarged fibres compared to contralateral TA muscles injected with AAV9‐miR‐Ctrl (Figure 5B), despite no significant changes in overall muscle weight (Figure S4A). Intramuscular injection of miR‐129‐3p decreased the protein levels of PARP1 and TRIM63 (Figure 5C) and resulted in higher levels of NAD^+^ (Figure 5D). In addition, mitochondrial DNA content was dramatically higher. We found an accumulation of ATP content in miR‐129‐3p‐overexpressing muscles. To evaluate mitochondrial activity in muscle fibres, we investigated the activity of succinate dehydrogenase (SDH), a key mitochondrial enzyme. The percentage of actual SDH‐positive Type I fibres in miR‐129‐3p‐infected muscles was 15% higher than that in contralateral muscles (Figures 5E and S4B). Transmission electron microscopy imaging revealed that the miR‐129‐3p‐infected muscles significantly exhibited enhanced mitochondrial contents and lowered abnormal mitochondria ratio (Figures 5F and S4C). Furthermore, miR‐129‐3p improved isometric force in obese mice, which exhibited higher twitch and tetanic force (Figure 5F). A recent study reported that the expression of miR‐129‐3p was downregulated in the muscle tissues of patients with obesity (Figure S5A, GSE 99891), supporting our results that miR‐129‐3p might have a crucial role for maintaining muscle health in obesity. Additionally, we found that miR‐129‐3p was downregulated in aged muscle, immobile muscle, nerve‐crushed muscle and muscle that had received BOTOX injections (Figure S5B–E). To evaluate the therapeutic potential of miR‐129‐3p, we examined its effects on muscle atrophy using a BOTOX‐induced mouse model. Notably, miR‐129‐3p significantly ameliorated the muscle weight loss induced by the BOTOX‐mediated muscle atrophy (Figure S5F–H). The attenuated weight loss in the TA muscles treated with miR‐129‐3p was accompanied by a greater CSA (Figure S5I–K). Taken together, our results suggest that exercise‐induced miR‐129‐3p might be a potential target to improve muscular deterioration, as shown in various muscle atrophy models.

### 3.6 miR‐129‐3p Regulates Mitochondrial Function in Human Skeletal Muscle Myoblasts (HSMMs)

**FIGURE 6:**
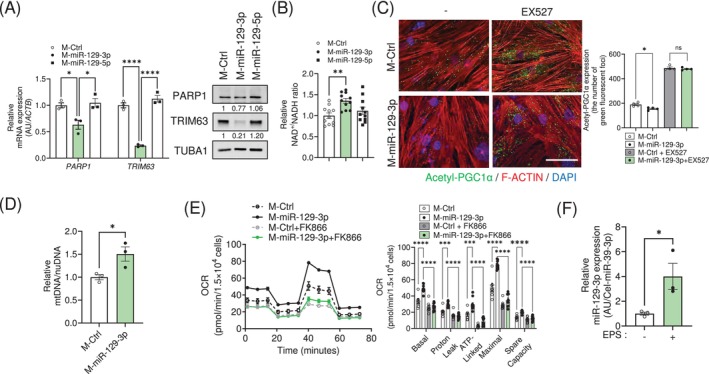
miR‐129‐3p enhances mitochondrial function in HSMMs. Human skeletal muscle myoblasts (HSMMs) were transfected with M‐miR‐129‐3p or M‐Ctrl. After 24 h, (A) (*left*) relative mRNA expression of *PARP1* and *TRIM63*. The data were normalized to *ACTB*. (*Right*) Protein levels of PARP1 and TRIM63. The data were normalized to TUBA1. (B) Intracellular NAD^+^/NADH ratio (*n* = 11). (C) (*Left*) Representative images of PLA. *Green*, Acetyl‐PGC1α; *Red*, F‐actin; *Blue*, DAPI. Scale bar, 100 μm. (*Right*) Quantification of green fluorescent foci (*n* = 4). (D) Relative mtDNA contents (*n* = 3). (E) OCR analysis in HSMMs. (F) Relative expression of miR‐129‐3p in EPS‐treated HSMM myotubes (*n* = 3). The data are presented as the mean ± SEM. *p < 0.05, **p < 0.01, ***p < 0.001, ****p < 0.0001. Statistical significance was assessed by Student's *t*‐test (for D and F), one‐way ANOVA (for A and B) or two‐way ANOVA (for C and E).

Since the miR‐129‐3p seed sequence is conserved in human *PARP1* 3′ UTR and *TRIM63* exon 7 (Figure S6A), we tested whether miR‐129‐3p could reduce human *PARP1* and *TRIM63* expression levels as well. Consistent with the results obtained in mice, M‐miR‐129‐3p transfection downregulated the mRNA and protein levels of PARP1 and TRIM63 (Figure 6A), showing enlarged diameters in the miR‐129‐3p overexpressing myotubes differentiated from HSMMs (Figure S6B). NAD^+^ content was significantly accumulated in myotubes transfected with M‐miR‐129‐3p (Figure 6B). SIRT1 was activated in those myotubes, as the number of positive fluorescent foci of acetylated PGC1α was significantly lowered (Figure 6C). Mitochondrial DNA content was significantly greater (Figure 6D). Consistent with the greater mitochondrial content, OCR were enhanced in the M‐miR‐129‐3p‐transfected HSMMs. This enhancement was abolished upon treatment with FK866 (Figure 6E) or SR18292 (Figure S6C). The OCR data was normalized to cell number since miR‐129‐3p did not affect cellular proliferation (Figure S6D). Interestingly, the expression of miR‐129‐3p was dramatically upregulated in EPS‐treated myotubes, suggesting that miR‐129‐3p could be induced by the contraction of myotubes (Figure 6F). As expected, *PARP1* and *TRIM63* expression levels were downregulated in EPS‐treated myotubes (Figure S6E). Overall, our data using HSMMs corroborate our findings in mice, and strengthen our confidence in miR‐129‐3p as a valuable agent for improving mitochondrial function.

## 4 Discussion

**FIGURE 7:**
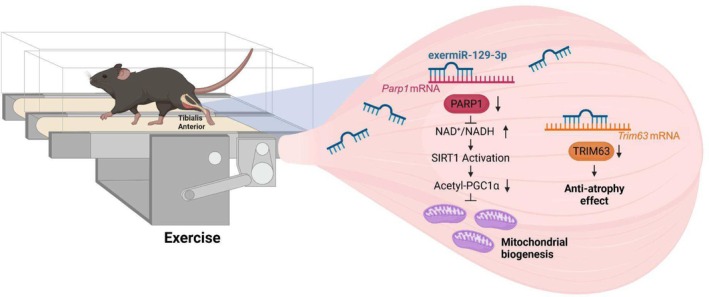
Graphical summary of how exermiR‐129‐3p regulates mitochondrial biogenesis and muscular hypertrophy.

Exercise is a valuable method to reduce skeletal muscle degeneration caused by obesity and other conditions [27]. Our study provides evidence that miR‐129‐3p is an exercise‐induced miRNA that regulates muscle physiology through PARP1‐SIRT1‐PGC1α axis. PARP1 is a multifunctional enzyme regulating various biological pathways such as DNA damage response, chromatin modification, cell death and inflammation [28, 29]. It has been shown previously that mice with either genetically ablated *Parp1* or pharmacologically inhibited PARP1 exhibited greater endurance exercise ability by activating SIRT1 [15, 16]. However, the upstream regulator of PARP1 during exercise remains elusive. In the present study, we identified miR‐129‐3p as an upstream regulator of PARP1 in an exercise context, which provides a mechanistic framework for understanding why PARP1 inhibition leads to greater endurance exercise ability. Taken together, our findings suggest that miR‐129‐3p has a crucial role in improving mitochondrial activity and muscle atrophy (Figure 7), underscoring its potential target as an exercise mimetic to maintain muscle health.

Our gene ontology (GO) analysis demonstrated that miR‐129‐3p modulates multiple biological processes beyond its direct targets. Since TRIM63 inhibition has an effect on the chemically induced muscle atrophy [30], rather than normal conditions without atrophic signalling, the biological processes, such as actin filament organization [31], positive regulation of myoblast differentiation [32] and JNK cascade [33], might be involved in the myotube size regulation, together with the inhibition of *Trim63* expression by miR‐129‐3p. Intriguingly, overexpressing miR‐129‐3p in muscle cells resulted in upregulation of genes related to synapse formation and cell junctions in Figure S1G, such as *Syne1*, *Schip1*, *Sntb1*, *Dbh* and *Sv2b*, implying the possibility of improving *de novo* neuromuscular junction formation. Previous reports have shown that muscles from exercised mice have significantly larger nerve terminals [34, 35]. Conversely, obese mice exhibited a decrease in synaptic areas, density and the number of nicotinic acetylcholine receptors [36]. Therefore, miR‐129‐3p might act as a pleiotropic regulator of muscle physiology, influencing the function of multiple processes such as neuromuscular junction formation and maintenance.

Although transgenic mice overexpressing miR‐129‐3p showed enhanced energy expenditure, we observed no significant changes in their body weight. This seemingly paradoxical finding led us to hypothesize that miR‐129‐3p overexpression may have induced changes in body composition, potentially reducing fat mass while promoting muscle hypertrophy, thus maintaining stable body weight despite increased energy expenditure. We acknowledge that a key limitation of our current study is the lack of detailed body composition analysis (e.g., MRI, DEXA or tissue dissection) to validate this hypothesis. Future studies incorporating these measurements will be essential to fully understand miR‐129‐3p's impact on body composition and metabolism.

Besides its cell‐autonomous role in the muscle, a recent study has demonstrated elevated expression of miR‐129‐3p in exosomes obtained from elderly individuals who exercise regularly [37]. The activity of PARPs, the main cellular NAD^+^ consumers, increases in the subcutaneous adipose tissue of larger monozygotic twins with a higher BMI [38]. PARP1 activation has been observed in both mice and patients with nonalcoholic fatty liver disease [39]. miR‐129‐3p may therefore have a beneficial effect on other metabolic tissues, such as the adipose tissue and the liver, through endocrine pathways mediated by exosomes. Since our study primarily focused on the intrinsic mechanism of how miR‐129‐3p, an exercise‐induced miRNA in skeletal muscle, regulates mitochondrial activity as well as muscle atrophy, further studies are needed to test whether miR‐129‐3p and potentially other exermiRs could mediate the systemic beneficial effects of physical exercise.

Collectively, we discovered that miR‐129‐3p is a miRNA induced by exercise that affects a range of genes, including *Parp1* and *Trim63*. Through its effects on the PARP1 signalling axis, miR‐129‐3p affects muscle physiology and health. Importantly, we showed that introducing miR‐129‐3p into an unhealthy muscle restores its function. Our results suggest that exermiR could act as an exercise mimetic to restore and maintain muscle health in a wide range of diseases and conditions where exercise is not feasible.

## Conflicts of Interest

K.‐S.K. is Aventi Inc.'s CEO. Conflicts of interest are not disclosed by the other authors.

## Supporting information


**Table S1** List of differentially expressed miRNAs in Ctrl vs. exercised mouse muscle.

**Table S2** List of differentially expressed miRNAs in Ctrl vs. EPS model.

**Table S3** List of differentially expressed genes in miR‐129 overexpressed C2C12 myotubes.

**Table S4** List of putative targets using TargetScan.

**Table S5** List of differentially expressed miRNAs in WT vs. *ob/ob* mouse muscle.

**Table S6** List of primer sequences.

**Figure S1** Exercise‐induced miR‐129‐3p modulates diverse biological processes in skeletal muscle. C57BL6 mice were exercised over a period of 4 weeks. (A) Exercised mice exhibited longer running times than control mice. (B) Heatmap of differentially expressed genes (DEGs) in C2C12 myotubes transfected with M‐miR‐129‐3p treatment compared with M‐Ctrl. Nine hundred twenty‐nine genes were upregulated, and 711 genes were downregulated. Z‐scores are shown. DEGs with *p* < 0.05 were used*.* (C) Relative *Parp1* mRNA expression (*n* = 6) in EPS‐treated C2C12 myotubes. The mRNA level was normalized to *Gapdh*. (D) Representative immunofluorescence images in C2C12 myotubes transfected with M‐miR‐129‐3p or I‐miR‐129‐3p. (*Top*) M‐miR‐129‐3p overexpression induced hypertrophy in C2C12 myotubes, whereas (*bottom*) its inhibition caused atrophy. These images quantified the diameter distribution and average of MyHC‐positive myotubes. *Green*, MyHC; *blue*, DAPI. Scale bars, 50 μm. (E) (*Left*) RNA sequencing analysis was presented for the expression of muscle‐specific E3 ligases (*Atrogin‐1* and *Trim63*) in C2C12 myotubes transfected with M‐miR‐129‐3p or M‐Ctrl. These data were presented by Z‐scores. (*Right*) Relative mRNA expressions of *Trim63* in C2C12 myotubes (*n* = 3). These mRNA expressions were normalized to *Actb*. (F) HEK 293T cells were transfected with firefly luciferase reporter constructs containing WT *Trim63* exon 7 or mutant *Trim63* exon 7 with deletion of the seed sequence (positions 961–984). Relative activity of luciferase was decreased in miR‐129‐3p co‐transfected cells and restored in mutant *Trim63* exon 7. (G) Gene Ontology (GO) biological process (BP) analysis was performed using DEGs with *p < 0.005* using Database for Annotation, Visualization and Integrated Discovery (DAVID), and the results were presented as a dot plot indicating Gene Ratio, Gene count and *p*‐value. Heatmap of DEGs associated with ‘Lipid homeostasis’ (10 genes), ‘Actin filament organization’ (8 genes), ‘Myoblast differentiation’ (5 genes), ‘Mitochondrial organization’ (8 genes) and ‘Neuronal regulation’ (13 genes). Z‐scores are shown. The data are presented as the mean ± SEM. *p < 0.05, **p < 0.01, ***p < 0.001*.* Statistical significance was assessed by Student’s *t*‐test (for C and D), Mann–Whitney test (for A) or one‐way ANOVA (E and F).
**Figure S2.** miR‐129‐3p enhances mitochondrial respiration in C2C12 myotubes. (A) Immunoblot analysis of the indicated proteins (SIRT1, Acetyl‐p53, p53, and TUBA1) in C2C12 myotubes. Overexpression of M‐miR‐129‐3p leads to decreased acetylation of p53. The protein abundance of SIRT1 and p53 was normalized to TUBA1. Acetyl‐p53 levels were normalized to p53. These levels were quantified using ImageJ software. (B) Oxygen consumption rates (OCR) were measured using the Seahorse XFe96 analyser. SR‐18292, a PGC1α inhibitor, was used to reduce PGC1α activity. (C) Cellular proliferation following miR‐129‐3p mimic transfection was assessed by CCK‐8 (*left*) and direct cell counting (*right*) (*n* = 6). The data are presented as the mean ± SEM. *p < 0.05, **p < 0.01, ****p < 0.0001. Statistical significance was assessed by two‐way ANOVA (for B and C).
**Figure S3.** Characterization of mice with muscular overexpression of miR‐129‐3p. (A) Average of TA muscle weight (mg/bw, *n* = 5) in mice injected with AAV9‐Ctrl or AAV9‐miR‐129‐3p. (B) Immunoblots of the indicated proteins (SIRT1, Acetyl‐p53, and p53) in TA muscle intramuscularly injected with either AAV9‐Ctrl or AAV9‐miR‐129‐3p (*n* = 3). Acetyl‐p53 levels were normalized to p53 and quantified using ImageJ software. (C) Measurement of twitch force in those muscles (*n* = 5). All force measurements were normalized to muscle weight (g) to account for differences in muscle mass. (D) qRT‐PCR analysis of miR‐129‐3p (*n* = 4) and immunoblots of GFP protein (*n* = 3) were performed to validate the overexpression of miR‐129‐3p and GFP in hindlimbs following tail vein injection with AAV‐lsl‐GFP‐miR‐129‐3p. (E) The respiratory exchange ratio has not changed (*n* = 4) in those muscles. In the same condition, (F) body weight and (G) grip strength of Myl1‐Cre mice were not changed during 2–4 weeks after injection (WT, *n* = 9; Myl1‐Cre, *n* = 11). The data are presented as the mean ± SEM. **p < 0.01*.* Statistical significance was assessed by Student’s *t*‐test (for A, C and D) or two‐way ANOVA (F and G).
**Figure S4.** SDH staining and TEM images of AAV9‐Ctrl or AAV9‐miR‐129‐3p‐injected muscles of obese mice. (A) Average of TA muscle weight (mg/bw, *n* = 4) in *ob/ob* mice injected with AAV9‐Ctrl or AAV9‐miR‐129‐3p. (B) (*Top*) Representative images of SDH staining. Red squares in images indicate the area of (*bottom*) the expanded images. Scale bars, 200 μm. (C) Representative images of TEM. Red squares indicate the regions magnified in Figure 5F. Scale bars, 1 μm. Quantification of abnormal mitochondria ratio relative to total mitochondria. The data are presented as the mean ± SEM. ***p < 0.001*.* Statistical significance was assessed by Student’s *t*‐test (for A and C).
**Figure S5.** miR‐129‐3p ameliorates the BOTOX‐mediated muscle atrophy. (A) Expression of miR‐129‐3p tends to downregulate in the muscles of obese human patients (GSE 99891) (*n* = 2). (B‐E) miR‐129‐3p expression levels are decreased under muscle atrophic conditions in mice, including (B) aging (young, 3‐month‐old; aged, 24‐month‐old, *n* = 6), (C) disuse (Ctrl, *n* = 3; disuse, *n* = 4), (D) nerve crush (*n* = 4) and (E) BOTOX injection (5 U/kg, *n* = 4). All miR‐129‐3p expression levels were normalized to the spike‐in control cel‐miR‐39‐3p. (F) Schematic diagram of AAV9‐miR‐129‐3p or AAV9‐Ctrl intramuscular injections into the TA muscle of BOTOX‐injected mice. Changes in (G) body weight and (H) TA weight (% changes to WT). (I) Representative images of muscle sections immunostained with Laminin (red) and DAPI (blue). Scale bar, 50 μm. Quantification of (J) the average myofiber CSA and (K) its distribution. The data are presented as the mean ± SEM. *p < 0.05, **p < 0.01. ***p < 0.001, ****p < 0.0001. Statistical significance was assessed by Student’s *t*‐test (for B, C, D and E), one‐way ANOVA (H and J) or two‐way ANOVA (G and K).
**Figure S6.** miR‐129‐3p inhibits expression of *PARP1* and *TRIM63* in EPS‐treated HSMM myotubes. (A) (*Top*) the miR‐129‐3p binding site in mouse *Parp1* 3*′* untranslated region (UTR) (positions 684–689) is conserved in human *PARP1* 3′ UTR (positions 724–730). (*Bottom*) The miR‐129‐3p binding site in the mouse *Trim63* exon 7 (positions 961–984) is conserved in the human *TRIM63* exon 7 (positions 993–1016). (B) Representative immunofluorescence images in HSMM myotubes transfected with M‐miR‐129‐3p or M‐Ctrl. These images quantified the diameter distribution and average of MyHC‐positive myotubes. *Green*, MyHC; *blue*, DAPI. Scale bar, 100 μm. (C) Oxygen consumption rates (OCR) were measured using the Seahorse XFe96 analyser. SR‐18292, a PGC1α inhibitor, was used to reduce PGC1α activity. (D) Cellular proliferation following miR‐129‐3p mimic transfection was assessed by CCK‐8 (*left*) and direct cell counting (*right*) (*n* = 3). (E) Relative *PARP1* and *TRIM63* mRNA expression (*n* = 6) in EPS‐treated HSMM myotubes. The mRNA level was normalized to *GAPDH*. The data are presented as the mean ± SEM. *p < 0.05, **p < 0.01. ***p < 0.001, ****p < 0.0001. Statistical significance was assessed by Student’s *t*‐test (for B (*right*) and E) or two‐way ANOVA (B (*mid*), C and D).
