# Supplementary material for: ExermiR‐129‐3p Enhances Muscle Function by Improving Mitochondrial Activity Through PARP1 Inhibition
Source: J Cachexia Sarcopenia Muscle. 2025 Apr 20;16(2):e13823. doi: 10.1002/jcsm.13823 (PMC12010049; doi:10.1002/jcsm.13823)
Supplement: Supplementary file 6 — Table S6 List of primer sequences. [file JCSM-16-e13823-s006.docx]

**Supporting Information for**

**ExermiR-129-3p enhances muscle function by improving mitochondrial activity through PARP1 inhibition**

Yeo Jin Shin^1,8,*^, Jae Won Yang^1,2,8^, Heeyeon Jeong^1,2^, Joyeong Kim^1,3^, Bora Lee^1^, Ji-Won Kim^1^, Seung-Min Lee^1^, Ju Yeon Kwak^1,4^, Young Hoon Son^1,5^, Kap Jung Kim^6^, Yong Ryoul Yang^1,2^, Chuna Kim^1,3^, Ki-Sun Kwon^1,7,*^, and Kwang-Pyo Lee^1,2,*^

^*^To whom correspondence: Aging Convergence Research Center, Korea Research Institute of Bioscience and Biotechnology (KRIBB), Daejeon 34141, Republic of Korea.

Kwang-Pyo Lee. Tel.: +82-42-860-4146. Fax: +82-42-879-8596. E-mail: [kplee@kribb.re.kr](mailto:kplee@kribb.re.kr),

Ki-Sun Kwon. Tel.: +82-42-860-4143. Fax: +82-42-879-8596. E-mail: [kwonks@kribb.re.kr](mailto:kwonks@kribb.re.kr) or

Yeo Jin Shin. Tel.: +82-42-860-4144. Fax: +82-42-879-8596. E-mail: [yjshin@kribb.re.kr](mailto:yjshin@kribb.re.kr)

**Materials and methods**

**Cell culture**

Human primary myoblasts, 3 to 4 passages, were utilized from a previous study, and these cells were isolated from paraspinal muscle tissue obtained from a 48-year-old male donor (IRB number: EMC 2018-06-003, P01-20170831-004)^1^. In summary, the muscle tissue was finely minced with scissors and then incubated in dissociation buffer containing Dispase (2.4 U/ml, Roche), Collagenase D (1.5 U/ml, Roche), and 2.5 mM CaCl_2_ at 37 °C for 45 minutes. Following incubation, the mixture was gently triturated with a serological pipette and filtered through a 40-μm nylon mesh (BD Biosciences) to remove any debris. The separated cells were subsequently collected and suspended in Ham's F-10 (Gibco) growth medium, which contained 20% FBS, amphotericin B/penicillin/streptomycin, and 5 ng/ml basic fibroblastic growth factor. C2C12 cells (American Type Culture Collection), 3 to 6 passages, were cultured in Dulbecco's Modified Eagle Medium (DMEM) with antibiotics and 10% Fetal Bovine Serum. Differentiation was induced 24 to 48 hours after seeding by replacing the growth medium with differentiation medium consisting of DMEM supplemented with antibiotics and 2% FBS. For miRNA transfection in myotubes, both mimics and inhibitors of miRNAs were procured from mirVana (Invitrogen), while small interfering RNAs (siRNAs) were acquired from Santa Cruz Biotechnology. Mimics, inhibitors of miRNA, and siRNA (ranging from 12.5 to 100 nM each) were transfected into either human primary myoblasts or C2C12 cells using RNAiMAX (Invitrogen), following the manufacturer’s recommended protocols. For the cellular proliferation assay, C2C12 cells or human primary myoblasts transfected with miRNA mimics were cultured for 72 hours followed by Cell Counting Kit-8 (CCK-8, Dojindo) assay according to the manufacturer's recommended protocols.

**Electrical pulse stimulation-induced *in vitro* exercise protocol**

We followed a previously reported protocol for *in vitro* exercise ^2^. Differentiated myotubes in 6-well plates underwent electrical pulse stimulation (EPS) at 37 °C using a C-PACE EP culture pacer (IonOptix, Ireland) for both mouse and human cells. EPS was administered as a 1 ms pulse stimulus with a 2 ms duration at 11.5 volts and 1 Hz frequency for 24 hours, followed by 1 hour of rest. Myotubes were collected immediately following the final contractile stimulus.

**Bioinformatic analysis of miRNA expression using captured probes**

The nCounter® miRNA Expression Assays kit from Nanostring (Seattle, WA, USA) was employed to analyze miRNA expression levels. A microRNA tag ligation reaction was performed, followed by hybridization of tagged microRNAs with the mouse microRNA code set at 65°C for 18 hours, according to the manufacturer’s protocol. The nCounter Digital Analyzer quantified specific target molecules by counting individual fluorescent barcodes. Normalization was performed per the manufacturer’s instructions, using Cel-miR-39-3p as the control. Data collection utilized a CCD camera to capture images of immobilized fluorescent reporters in the sample cartridge. MiRNA data analysis was performed using the nSolver (version 4.0) software analysis and represented their expression change exceeded a significance threshold (*P < 0.05*).

**Transcriptome analysis**

Total RNA was utilized to prepare libraries using the TruSeq™ Stranded Total RNA Library Prep Kit (Illumina, San Diego, CA, USA). Following the manufacturer’s guidelines, mRNA was employed for cDNA synthesis and fragmentation. Illumina indices 1–12 were used for indexing, and PCR was conducted for enrichment. The libraries were subsequently analyzed with the Agilent D1000 ScreenTape System to determine the average fragment size. Quantification was carried out with the Library Quantification Kit and StepOne™ Real-Time PCR System (Life Technologies, Carlsbad, CA, USA). The NovaSeq 6000 platform (Illumina, San Diego, CA, USA) was used to high-throughput sequencing of paired-end reads. A total of 929 upregulated and 711 downregulated genes were selected based on p-values ranked in ascending order. Upregulated genes had a fold change greater than 1.5, while downregulated genes had a fold change of less than 0.5. These selected genes were used for functional annotation analysis.

**Functional Annotation Analysis**

Gene Ontology (GO) Biological Process (BP) enrichment analysis was performed using the Database for Annotation, Visualization, and Integrated Discovery (DAVID) v.2021 (<https://david.ncifcrf.gov>). Differentially expressed genes (DEGs, *P < 0.05*) in M-miR-129-3p compared to M-Ctrl were used for separate enrichment analyses of upregulated and downregulated genes. Only significantly enriched GO:BP terms (*P < 0.05*) were included in the results.

**3’UTR-binding luciferase assay**

Luciferase assays were conducted following the protocol described in a previous study ^3^. The full-length 723 nucleotide 3′ UTR of mouse *Parp1* mRNA or the exon 7 (125 nucleotides, positions 959-1083) of mouse *Trim63* was inserted into the pmirGLO vector (Promega), housing the *Luc2* coding sequence in the multi-cloning site, while the hRluc-neo coding sequence served as an internal control. Additionally, a *Parp1* 3′ UTR mutant lacking the miR-129-3p binding region (positions 684-689) or a Trim63 exon 7 mutant lacking the miR-129-3p binding region (positions 975-981) was cloned into the pmirGLO vector for luciferase assays. Transfection of 293T cells was carried out using Lipofectamine 2000 (Invitrogen) with 50 nM of miRNA mimic and 200 ng of luciferase plasmids. Luciferase activity was measured 48 hours post-transfection using the Dual-Luciferase Reporter Assay System (Promega) and Victor X3 (Perkin Elmer).

**Proximity ligation assay**

Cells were fixed and permeabilized as described for immunostaining and used to perform PLA using NaveniFlex XR (Navinci, Sweden) according to the manufacturer’s instructions. Briefly, cells were first incubated with a commercial blocking buffer, followed by overnight incubation at 4 °C with primary antibodies (anti-PGC1; Millipore; ST1202, anti-acetyl-lysine; CST; 9441) diluted in the commercial solution at specified concentrations. Following PLA probe incubation, ligation, and amplification steps, samples were additionally stained with rhodamine phalloidin to visualize actin structures. Finally, samples were mounted with Antifade Mounting Medium containing DAPI (Vector Laboratories) for nuclear staining. Images were captured using a Nikon Eclipse Ti-U microscope. For the quantification of PGC1α acetylation, four random views were selected, and the spots were counted.

**Muscle atrophy mouse models**

Young (3-month-old) and aged (24-month-old) C57BL/6 mice were purchased from the Laboratory Animal Resource Center (Korea Research Institute of Bioscience and Biotechnology, KRIBB). In the disused muscle atrophy model, muscles were surgically anchored using staples. Muscle samples were then collected ten days post-injury for analysis. In the nerve crush-induced muscle atrophy model, the sciatic nerve was crushed for 10 seconds, followed by suturing. The contralateral leg received only sham surgery without a crush injury. Muscle samples were then collected seven days post-injury for analysis. In the BOTOX-induced muscle atrophy model, TA muscles were injected with BOTOX (5 U/kg, Allergan), whereas contralateral TA muscles were injected with PBS, and then collected seven days post-injury for analysis. To examine the therapeutic effect of miR-129-3p in this model, intramuscular injection of AAV9-Ctrl or AAV9-miR-129-3p was performed seven days prior to BOTOX administration.

**Quantitative PCR analysis of mRNA and miRNA expression**

RNA isolation and cDNA synthesis were conducted following standard protocols. The StepOnePlus™ system from Applied Biosystems was used to perform quantitative reverse transcription-PCR (qRT-PCR) in a 20 μL reaction volume. The reaction mixture included cDNA, primers, and SYBR Master Mix. The primer sequences are provided in Table S6. Data was normalized according to the mRNA levels of *Actb* or *Gapdh* in each reaction. TaqMan Advanced miRNA assays were used to analyze mature miRNA expression, following the manufacturer's procedure (Applied Biosystems). TaqMan Universal PCR Master Mix II (without uracil N-glycosylase) and TaqMan Small RNA Assay Mix were used to perform qRT-PCR in 96-well plates. Specific TaqMan probes were used to detect the expression of miR-129-3p (Catalog number: A25576, Assay ID: mmu480873_mir) and Cel-miR-39-3p (Catalog number: A25576, Assay ID: 478293_mir). Cel-miR-39-3p was utilized as the endogenous control.

**Western blotting**

Muscle tissues and myoblasts were lysed in a buffer consisting of 50mM Tris-Cl (pH 7.4), 150mM NaCl, 0.5% Triton X-100, 1mM EDTA, and 1mM MgCl_2_, supplemented with protease and phosphatase inhibitors. Following homogenization, the lysates were centrifuged at 15,000 x g for 20 minutes at 4 °C. The resulting supernatants were analyzed by SDS polyacrylamide gel electrophoresis, followed by immunoblotting. Immunoblot analysis utilized antibodies targeting specific proteins, including PARP1 (Cell Signaling Technology), TUBA1 (α-tubulin; Santa Cruz Biotechnology), ACTB (β-actin; Abcam), ACTN1 (Santa Cruz Biotechnology), TRIM63 (ECM Biosciences), SIRT1 (Cell Signaling Technology), Acetyl-p53 (Thermo Scientifics), p53 (Cell Signaling Technology), VCL (Cell Signaling Technology), and GFP (Abcam). GAPDH was detected using a specific antibody produced in our laboratory ^4^.

**Measurement of NAD contents**

For the measurement of NAD contents, we employed the NAD/NADH-Glo™ assay kit from Promega following the manufacturer’s protocol. Briefly, myoblasts were lysed using PBS + Base solution, and equal volumes of lysates were separated. To measure NAD^+^ levels, one portion of the lysates was added to 0.4 M HCl and incubated at 60 °C for 15 minutes. Another portion of the lysates was incubated at 60 °C for 15 minutes and then added to the HCl/Trizma solution for NADH measurement. Finally, NAD^+^ and NADH levels were determined using a luciferase reaction after addition to the NAD^+^/NADH-Glo solution, and measurements were taken using Victor X3 (Perkin Elmer). For measurement of NAD^+^ contents in muscle tissue, muscle samples were homogenized using a PBS + Base solution. The lysates were then deproteinized using a 10 kDa spin column (Abcam) and proceeded with the protocol for NAD^+^ content measurement similar to that used for cells.

**Measurement of ATP contents**

We followed the manufacturer's protocols (Abcam, ab83355) for the measurement of ATP in muscle. Muscle lysates were deproteinized using TCA (Abcam, ab204708). Deproteinized muscle lysates and standards were added to the 96-well assay plate, followed by the addition of the reaction mix, and then incubated for 30 minutes at room temperature. Subsequently, ATP levels were analyzed using Victor X3.

**Measurement of mtDNA contents**

For measurement of mtDNA contents, gDNA preparation followed standard protocols ^5^. Ten ng of DNA was used for qPCR with a pair of primers for mtDNA (*MT-CO1*) and *Lpl* for normalization, as described before. The primer sequences are provided in Table S6.

**Measurement of Oxygen Consumption Rates**

OCR was measured using the Seahorse XFe96 analyzer (Agilent). The calibration plate was equilibrated overnight in the CO2-free incubator before being transferred to the Seahorse XFe96 analyzer. Compound solutions (oligomycin, carbonyl cyanide-4-(trifluoromethoxy) phenylhydrazone (FCCP), rotenone/antimycin A) were loaded into each port on the sensor cartridge, and the calibration step was performed. Differentiated myoblasts were replaced with XF Assay Medium supplemented with 1 mM pyruvate, 5 mM D-glucose, and 4 mM L-glutamine (pH 7.4). To abolish the effect of M-miR-129-3p, myotubes were treated with FK866 (50 nM) for 2 hours or SR-18292 (50 μM) for 6 hours before OCR measurements. These cells were allowed to equilibrate for 30 minutes in a CO_2_-free incubator at 37 °C. Cell plates were then loaded into the analyzer after the calibration step was completed. Measurements of OCR were taken at baseline and following sequential injections of (a) oligomycin (1.5 μM), (b) FCCP (0.5 μM for C2C12 myotubes and 2 μM for human primary myotubes), and (c) rotenone/antimycin A (0.5 μM), respectively.

**Immunostaining and immunohistochemistry**

For immunostaining, we referred to the protocol previously reported in our study ^6^. First, fixed C2C12 or HSMM myotubes were treated with 4% paraformaldehyde and 0.3% Triton X-100 to enhance permeability. Subsequently, they were blocked in a solution containing 3% bovine serum albumin in PBS and then incubated with anti-MyHC antibodies (Santa Cruz Biotechnology). After PBS washing steps, the samples were exposed to secondary antibodies labeled with AlexaFluor 488 (Invitrogen). For immunohistochemical examination of skeletal muscle tissue, fixation was performed using 4% paraformaldehyde. Frozen sections, each 10 μm thick, underwent staining with antibodies using standard protocols. The antibody used targeted Laminin (Sigma-Aldrich). Finally, samples were mounted with Antifade Mounting Medium containing DAPI (Vector Laboratories) for nuclear staining. Images were captured using a Nikon Eclipse Ti-U microscope. To determine the cross-sectional area, six random fields of view were selected, and measurements were taken using microscope imaging software (NIS-Elements Basic Research, Nikon).

**Measurement of muscle-specific force *ex vivo***

We measured muscle-specific force according to a previously reported protocol ^3, 4^. Intact TA muscle-tendon complexes were isolated from mice and vertically mounted in a bath chamber filled with carbogen (95% O2/5% CO2)-saturated Krebs-Ringer buffer containing 118 mM NaCl, 4.75 mM KCl, 24.8 mM NaHCO_3_, 1.18 mM KH_2_PO_4_, 2.5 mM CaCl_2_ 2H_2_O, 1.18 mM MgSO_4_, and 10 mM glucose, adjusted to pH 7.4 and maintained at 25 °C. The muscle was clamped to the bottom of the organ bath, and the tendon was attached to a force transducer (AD Instruments, USA) via a string. The optimal muscle length (L0) was determined using a modified previous protocol that elicited the highest twitch force. Twitch forces (mN) were recorded using an electrical stimulator (AD Instruments, USA) at 100 V and 1 Hz, while tetanus forces (mN) were measured at frequencies ranging from 10 to 200 Hz. Isometric forces were normalized to the weight of the TA muscle. All experiments were conducted at room temperature (25 °C) and analyzed using LabChart software (AD Instruments, USA).

**Succinate dehydrogenase assay**

We followed a previously reported protocol for SDH staining ^7^. Briefly, fresh muscle was frozen in an OCT compound within a mold. Using a cryostat, skeletal muscle cross-sections (10 μm thick) were sliced and mounted onto positively charged glass slides. The slide with muscle sections was transferred into a chamber and incubated in a prewarmed Incubation Solution (50 mM Sodium succinate, 50 mM Phosphate buffer, 0.5 mg/ml nitroblue tetrazoliumand) at 37 °C for 60 minutes. After incubation, the samples were washed three times in distilled water for 1 minute each. The sections were then mounted using a glycerol-based mounting medium, and images were captured using a Nikon Eclipse Ti-U microscope. For quantification, three random fields of view were selected, and the number of SDH-positive fibers was calculated relative to the total number of fibers.

**Indirect calorimetry test**

We followed a previously reported protocol for indirect calorimetry test ^8^. The respiratory quotient (RQ) and the energy expenditure (EE) were measured at the end of the experiment. The monitoring system (Apparatus Oxylet System, Panlab/Harvard, Barcelona, Spain) assessed respiratory metabolism using indirect calorimetry, measuring oxygen consumption (VO_2_) and carbon dioxide generation (VCO_2_). The VO_2_ and VCO_2_ levels were corrected based on their body mass. The RQ, or VCO_2_/VO_2_ ratio, was used to assess fuel consumption and energy generation. Data were collected for 24 hours, and throughout the testing period, the mice had unlimited access to food and water. The area under the curve was used to determine the EE during a 24-hour period using GraphPad Prism software.

**Transmission electron microscope analysis**

We performed the EM processing following the previously reported ^9^. Muscle tissues were initially fixed in 2.5% glutaraldehyde in 0.1 M phosphate buffer (pH 7.4) for 2 hours at 4 °C, followed by post-fixing with 1% osmium tetroxide on ice for 2 hours. Subsequently, the muscle tissues were dehydrated in a series of ethanol and propylene oxides, and then embedded. Polymerization was carried out using pure resin at 70 °C for two days. Ultrathin sections (EM UC7, Leica, Germany, and installed at Korea Basic Science Institute) were obtained with an ultramicrotome (UltraCut-UCT, Leica, Austria) and collected on 100-mesh copper grids. After staining with 2 % uranyl acetate and lead citrate, the sections were analyzed by TEM (JEM-1400Plus, JEOL, Japan, and installed at Korea Basic Science Institute) at 120 kV.

**Data availability**

The datasets generated and/or analyzed during the current study are available in the Korea BioData Station [https://kbds.re.kr, BioProject ID: KAP240778] repository.

**Schematics and diagrams**

BioRender (Science Suite Inc.) was used to construct the schematics and diagrams.

**Tables**

**Table S1 (separate file).** List of differentially expressed miRNAs in Ctrl vs Exercised mouse muscle

**Table S2 (separate file).** List of differentially expressed miRNAs in Ctrl vs EPS model

**Table S3 (separate file).** List of differentially expressed genes in miR-129 overexpressed C2C12 myotubes

**Table S4 (separate file).** List of putative targets using TargetScan

**Table S5 (separate file).** List of differentially expressed miRNAs in WT vs *ob/ob* mouse muscle

**Table S6. List of primer sequences**

| Species | Primer | Sequence |
| --- | --- | --- |
| Mouse | *Parp1 sense* | TCG ATG GGA AAG TCC CAC AC |
|  | *Parp1 antisense* | GGC CGT CTT CTT GAC CTT CT |
|  | *Trim63 sense* | GTG TGA GGT GCC TAC TTG CTC |
|  | *Trim63 antisense* | GCT CAG TCT TCT GTC CTT GGA |
|  | *Ndusfa1 sense* | TGA TGG AAC GCG ATA GAC G |
|  | *Ndusfa1 antisense* | GCC AGG AAA ATG CTT CCT TA |
|  | *Sdhb sense* | CCT GCT CTG TGG TGG ACT ACT |
|  | *Sdhb antisense* | CCC ATG AAC GTA GTC GGT AAC |
|  | *Cytb sense* | CAT TTA TTA TCG CGG CCC TA |
|  | *Cytb antisense* | TGG GTT GTT TGA TCC TGT TTC |
|  | *Cox7a1 sense* | CGA AGA GGG GAG GTG ACT C |
|  | *Cox7a1 antisense* | AGC CTG GGA GAC CCC TAG |
|  | *Atp5i sense* | AAA ACT GGT AGC TTC AAA CAC CTT |
|  | *Atp5i antisense* | ACA ATG CCA CGT TTG CCT AT |
|  | *MT-CO1 sense* | ACC ATC ATT TCT CCT TCT CCT A |
|  | *MT-CO1 antisense* | TAG ATT TCC GGC TAG AGG TG |
|  | *Lpl sense* | TTT GTG AAA TGC CAT GAC AAG |
|  | *Lpl antisense* | CAG ATG CTT TCT TCT CTT GTT TGT |
|  | *Gapdh sense* | AGG TCG GTG TGA ACG GAT TTG |
|  | *Gapdh antisense* | TGT AGA CCA TGT AGT TGA GGT CA |
|  | *Actb1 sense* | GGC TGT ATT CCC CTC CAT |
|  | *Actb1 antisense* | CCA GTT GGT AAC AAT GCC ATG |
| Human | *PARP1 sense* | CCA AGC CAG TTC AGG ACC TCA T |
|  | *PARP1 sense* | GGA TCT GCC TTT TGC TCA GCT TC |
|  | *TRIM63 sense* | GAG GAT TCC CGT CGA GTG AC |
|  | *TRIM63 antisense* | CAG GGA CTG GAT GGC AGT TT |
|  | *NDUSFA1 sense* | GTT CGA GAT TCT CCC CGG AC |
|  | *NDUSFA1 antisense* | TGT GGA TGT ACG CAG TAG CC |
|  | *SDHB sense* | CAC TCT AGC TTG CAC CCG AA |
|  | *SDHB antisense* | ACA TGT GTG GAA GAG GGT AGA |
|  | *CYTB sense* | CCC ACC CCA TCC AAC ATC TC |
|  | *CYTB antisense* | GCG TCT GGT GAG TAG TGC AT |
|  | *COX7A1 sense* | GAA CCG CTT TCA GAA CCG AG |
|  | *COX7A1 antisense* | CCC TTC AGG TAC AAC GGG AT |
|  | *ATP5I sense* | TCT GCA GAC GCT TCT CC |
|  | *ATP5I antisense* | ACG ATA TTT CCG AGC ACA GAG |
|  | *MT-CO1 sense* | CGA AGA GGG GCG TTT GGT AT |
|  | *MT-CO1 antisense* | GCG TTT CCC CGC ATA AAC AA |
|  | *MRPS18C sense* | TGA GGA CCT GCC CAT TTC AA |
|  | *MRPS18C antisense* | GTG CCT TCC ATA AAT GCA TCC |
|  | *GAPDH sense* | TGTTGCCATCAATGACCC |
|  | *GAPDH antisense* | CCCACGACGTACTCAGCG |

**Figure Legends**

**Figure S1. Exercise-induced miR-129-3p modulates diverse biological processes in skeletal muscle.**

C57BL6 mice were exercised over a period of 4 weeks. (A) Exercised mice exhibited longer running times than control mice. (B) Heatmap of differentially expressed genes (DEGs) in C2C12 myotubes transfected with M-miR-129-3p treatment compared with M-Ctrl. 929 genes were upregulated, and 711 genes were downregulated. Z-scores are shown. DEGs with *P < 0.05* were used*.* (C) Relative *Parp1* mRNA expression (n = 6) in EPS-treated C2C12 myotubes. The mRNA level was normalized to *Gapdh*. (D) Representative immunofluorescence images in C2C12 myotubes transfected with M-miR-129-3p or I-miR-129-3p. (*Top*) M-miR-129-3p overexpression induced hypertrophy in C2C12 myotubes, whereas (*bottom*) its inhibition caused atrophy. These images quantified the diameter distribution and average of MyHC-positive myotubes. *Green*, MyHC; *blue*, DAPI. Scale bars. 50 μm. (E) (*Left*) RNA sequencing analysis was presented for the expression of muscle-specific E3 ligases (*Atrogin-1* and *Trim63*) in C2C12 myotubes transfected with M-miR-129-3p or M-Ctrl. These data were presented by Z-scores. (*Right*) Relative mRNA expressions of *Trim63* in C2C12 myotubes (n = 3). These mRNA expressions were normalized to *Actb*. (F) HEK 293T cells were transfected with firefly luciferase reporter constructs containing WT *Trim63* exon 7 or mutant *Trim63* exon 7 with deletion of the seed sequence (positions 961-984). Relative activity of luciferase was decreased in miR-129-3p co-transfected cells and restored in mutant *Trim63* exon 7. (G) Gene Ontology (GO) biological process (BP) analysis was performed using DEGs with *P < 0.005* using Database for Annotation, Visualization, and Integrated Discovery (DAVID), and the results were presented as a dot plot indicating Gene Ratio, Gene count, and *P*-value. Heatmap of DEGs associated with 'Lipid homeostasis' (10 genes), 'Actin filament organization' (8 genes), 'Myoblast differentiation' (5 genes), 'Mitochondrial organization' (8 genes), and 'Neuronal regulation' (13 genes). Z-scores are shown. The data are presented as the mean ± SEM. **P < 0.05, **P < 0.01, ***P < 0.001.* Statistical significance was assessed by Student’s t-test (for C and D), Mann-Whitney test (for A), or one-way ANOVA (E and F).

**Figure S2. miR-129-3p enhances mitochondrial respiration in C2C12 myotubes.**

(A) Immunoblot analysis of the indicated proteins (SIRT1, Acetyl-p53, p53, TUBA1) in C2C12 myotubes. Overexpression of M-miR-129-3p leads to decreased acetylation of p53. The protein abundance of SIRT1 and p53 was normalized to TUBA1. Acetyl-p53 levels were normalized to p53. These levels were quantified using ImageJ software. (B) Oxygen consumption rates (OCR) were measured using the Seahorse XFe96 analyzer. SR-18292, a PGC1α inhibitor, was used to reduce PGC1α activity. (C) Cellular proliferation following miR-129-3p mimic transfection was assessed by CCK-8 (*left*) and direct cell counting (*right*) (n = 6). The data are presented as the mean ± SEM. **P < 0.05, **P < 0.01, ****P < 0.0001*. Statistical significance was assessed by two-way ANOVA (for B and C).

**Figure S3. Characterization of mice with muscular overexpression of miR-129-3p.**

(A) Average of TA muscle weight (mg/bw, n = 5) in mice injected with AAV9-Ctrl or AAV9-miR-129-3p. (B) Immunoblots of the indicated proteins (SIRT1, Acetyl-p53, p53) in TA muscle intramuscularly injected with either AAV9-Ctrl or AAV9-miR-129-3p (n = 3). Acetyl-p53 levels were normalized to p53 and quantified using ImageJ software. (C) Measurement of twitch force in those muscles (n = 5). All force measurements were normalized to muscle weight (g) to account for differences in muscle mass. (D) qRT-PCR analysis of miR-129-3p (n = 4) and immunoblots of GFP protein (n = 3) were performed to validate the overexpression of miR-129-3p and GFP in hindlimbs following tail vein injection with AAV-lsl-GFP-miR-129-3p. (E) The respiratory exchange ratio has not changed (n = 4) in those muscles. In the same condition, (F) body weight and (G) grip strength of Myl1-Cre mice were not changed during 2-4 weeks after injection (WT, n = 9; Myl1-Cre, n = 11). The data are presented as the mean ± SEM. ***P < 0.01.* Statistical significance was assessed by Student’s t-test (for A, C, and D) or two-way ANOVA (F and G).

**Figure S4. SDH staining and TEM images of AAV9-Ctrl or AAV9-miR-129-3p-injected muscles of obese mice.**

(A) Average of TA muscle weight (mg/bw, n = 4) in *ob/ob* mice injected with AAV9-Ctrl or AAV9-miR-129-3p. (B) (*Top*) Representative images of SDH staining. Red squares in images indicate the area of (*bottom*) the expanded images. Scale bars, 200 μm. (C) Representative images of TEM. Red squares indicate the regions magnified in Figure 5F. Scale bars, 1 μm. Quantification of abnormal mitochondria ratio relative to total mitochondria. The data are presented as the mean ± SEM. ****P < 0.001.* Statistical significance was assessed by Student’s t-test (for A and C).

**Figure S5. miR-129-3p ameliorates the BOTOX-mediated muscle atrophy.**

(A) Expression of miR-129-3p tends to downregulate in the muscles of obese human patients (GSE 99891) (n = 2). (B-E) miR-129-3p expression levels are decreased under muscle atrophic conditions in mice, including (B) aging (young, 3-month-old; aged, 24-month-old, n = 6), (C) disuse (Ctrl, n = 3; Disuse, n = 4), (D) nerve crush (n = 4), and (E) BOTOX-injection (5U/kg, n = 4). All miR-129-3p expression levels were normalized to the spike-in control cel-miR-39-3p. (F) Schematic diagram of AAV9-miR-129-3p or AAV9-Ctrl intramuscular injections into the TA muscle of BOTOX-injected mice. Changes in (G) body weight and (H) TA weight (% changes to WT). (I) Representative images of muscle sections immunostained with Laminin (red) and DAPI (blue). Scale bar, 50 μm. Quantification of (J) the average myofiber CSA and (K) its distribution. The data are presented as the mean ± SEM. **P < 0.05, **P < 0.01. ***P < 0.001, ****P < 0.0001.* Statistical significance was assessed by Student’s t-test (for B, C, D, and E), one-way ANOVA (H and J), or two-way ANOVA (G and K).

**Figure S6**. **miR-129-3p inhibits expression of *PARP1* and *TRIM63* in EPS-treated HSMM myotubes.**

(A) (*Top*) the miR-129-3p binding site in mouse *Parp1* 3′ untranslated region (UTR) (positions 684-689) is conserved in human *PARP1* 3′ UTR (positions 724-730). (*Bottom*) The miR-129-3p binding site in the mouse Trim63 exon 7 (positions 961-984) is conserved in the human TRIM63 exon 7 (positions 993-1016). (B) Representative immunofluorescence images in HSMM myotubes transfected with M-miR-129-3p or M-Ctrl. These images quantified the diameter distribution and average of MyHC-positive myotubes. *Green*, MyHC; *blue*, DAPI. Scale bar. 100 μm. (C) Oxygen consumption rates (OCR) were measured using the Seahorse XFe96 analyzer. SR-18292, a PGC1α inhibitor, was used to reduce PGC1α activity. (D) Cellular proliferation following miR-129-3p mimic transfection was assessed by CCK-8 (*left*) and direct cell counting (*right*) (n = 3). (E) Relative *PARP1* and *TRIM63* mRNA expression (n = 6) in EPS-treated HSMM myotubes. The mRNA level was normalized to *GAPDH*. The data are presented as the mean ± SEM. **P < 0.05, **P < 0.01. ***P < 0.001, ****P < 0.0001.* Statistical significance was assessed by Student’s t-test (for B (*right*) and E) or two-way ANOVA (B (*mid*), C, and D).

**References**

1. Son YH, Kim WJ, Shin YJ, Lee SM, Lee B, Lee KP, et al. Human primary myoblasts derived from paraspinal muscle reflect donor age as an experimental model of sarcopenia. Exp Gerontol. 2023;181:112273.

2. Lee B, Kim SK, Shin YJ, Son YH, Yang JW, Lee SM, et al. Genome-wide analysis of a cellular exercise model based on electrical pulse stimulation. Sci Rep. 2022;12:21251.

3. Shin YJ, Kwon ES, Lee SM, Kim SK, Min KW, Lim JY, et al. A subset of microRNAs in the Dlk1-Dio3 cluster regulates age-associated muscle atrophy by targeting Atrogin-1. J Cachexia Sarcopenia Muscle. 2020;11:1336-50.

4. Lee SM, Lee SH, Jung Y, Lee Y, Yoon JH, Choi JY, et al. FABP3-mediated membrane lipid saturation alters fluidity and induces ER stress in skeletal muscle with aging. Nat Commun. 2020;11:5661.

5. Liu J, Liang X, Zhou D, Lai L, Xiao L, Liu L, et al. Coupling of mitochondrial function and skeletal muscle fiber type by a miR-499/Fnip1/AMPK circuit. EMBO Mol Med. 2016;8:1212-28.

6. Lee KP, Shin YJ, Panda AC, Abdelmohsen K, Kim JY, Lee SM, et al. miR-431 promotes differentiation and regeneration of old skeletal muscle by targeting Smad4. Genes Dev. 2015;29:1605-17.

7. Nachlas MM, Tsou KC, De Souza E, Cheng CS, Seligman AM. Cytochemical demonstration of succinic dehydrogenase by the use of a new p-nitrophenyl substituted ditetrazole. J Histochem Cytochem. 1957;5:420-36.

8. Cappelli AP, Zoppi CC, Barbosa-Sampaio HC, Costa JM, Jr., Protzek AO, Morato PN, et al. Taurine-induced insulin signalling improvement of obese malnourished mice is associated with redox balance and protein phosphatases activity modulation. Liver Int. 2014;34:771-83.

9. Ho DH, Je AR, Lee H, Son I, Kweon HS, Kim HG, et al. LRRK2 Kinase Activity Induces Mitochondrial Fission in Microglia via Drp1 and Modulates Neuroinflammation. Exp Neurobiol. 2018;27:171-80.
