# Supplementary material for: ExermiR‐129‐3p Enhances Muscle Function by Improving Mitochondrial Activity Through PARP1 Inhibition
Source: J Cachexia Sarcopenia Muscle. 2025 Apr 20;16(2):e13823. doi: 10.1002/jcsm.13823 (PMC12010049; doi:10.1002/jcsm.13823)
Supplement: Supplementary file 7 — Figure S1 Exercise‐induced miR‐129‐3p modulates diverse biological processes in skeletal muscle. C57BL6 mice were exercised over a period of 4 weeks. (A) Exercised mice exhibited longer running times than control mice. (B) Heatmap of differentially expressed genes (DEGs) in C2C12 myotubes transfected with M‐miR‐129‐3p treatment compared with M‐Ctrl. Nine hundred twenty‐nine genes were upregulated, and 711 genes were downregulated. Z‐scores are shown. DEGs with p < 0.05 were used. (C) Relative Parp1 mRNA expression (n = 6) in EPS‐treated C2C12 myotubes. The mRNA level was normalized to Gapdh. (D) Representative immunofluorescence images in C2C12 myotubes transfected with M‐miR‐129‐3p or I‐miR‐129‐3p. (Top) M‐miR‐129‐3p overexpression induced hypertrophy in C2C12 myotubes, whereas (bottom) its inhibition caused atrophy. These images quantified the diameter distribution and average of MyHC‐positive myotubes. Green, MyHC; blue, DAPI. Scale bars, 50 μm. (E) (Left) RNA sequencing analysis was presented for the expression of muscle‐specific E3 ligases (Atrogin‐1 and Trim63) in C2C12 myotubes transfected with M‐miR‐129‐3p or M‐Ctrl. These data were presented by Z‐scores. (Right) Relative mRNA expressions of Trim63 in C2C12 myotubes (n = 3). These mRNA expressions were normalized to Actb. (F) HEK 293T cells were transfected with firefly luciferase reporter constructs containing WT Trim63 exon 7 or mutant Trim63 exon 7 with deletion of the seed sequence (positions 961–984). Relative activity of luciferase was decreased in miR‐129‐3p co‐transfected cells and restored in mutant Trim63 exon 7. (G) Gene Ontology (GO) biological process (BP) analysis was performed using DEGs with p < 0.005 using Database for Annotation, Visualization and Integrated Discovery (DAVID), and the results were presented as a dot plot indicating Gene Ratio, Gene count and p‐value. Heatmap of DEGs associated with ‘Lipid homeostasis’ (10 genes), ‘Actin filament organization’ (8 genes), ‘Myoblast differenti [file JCSM-16-e13823-s001.pptx]

## Slide 1
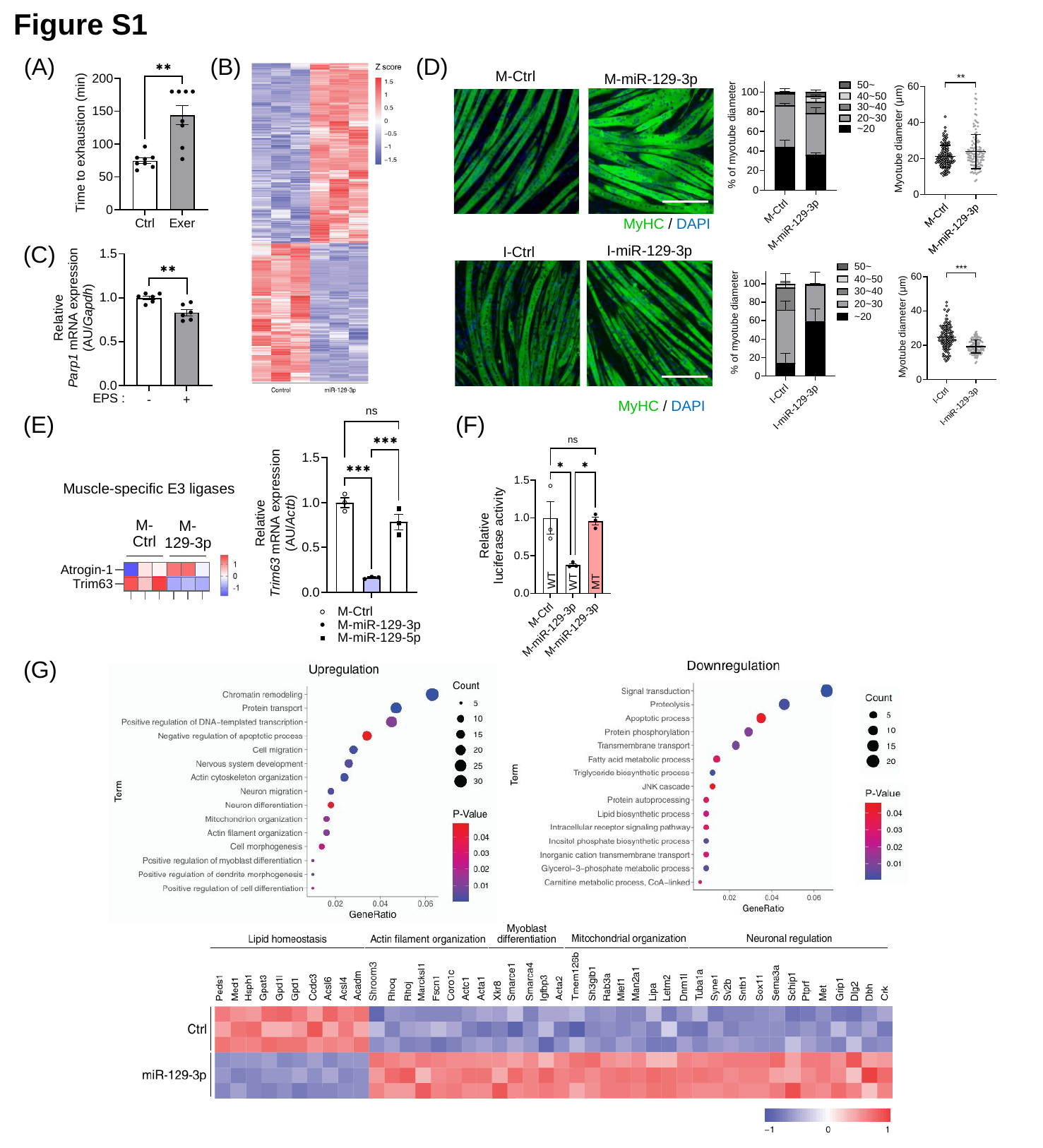

Figure S1
(A)
(B)
(D)
M-Ctrl
M-miR-129-3p
MyHC / DAPI
(C)
I-miR-129-3p
I-Ctrl
MyHC / DAPI
(E)
(F)
Muscle-specific E3 ligases
(G)

## Slide 2
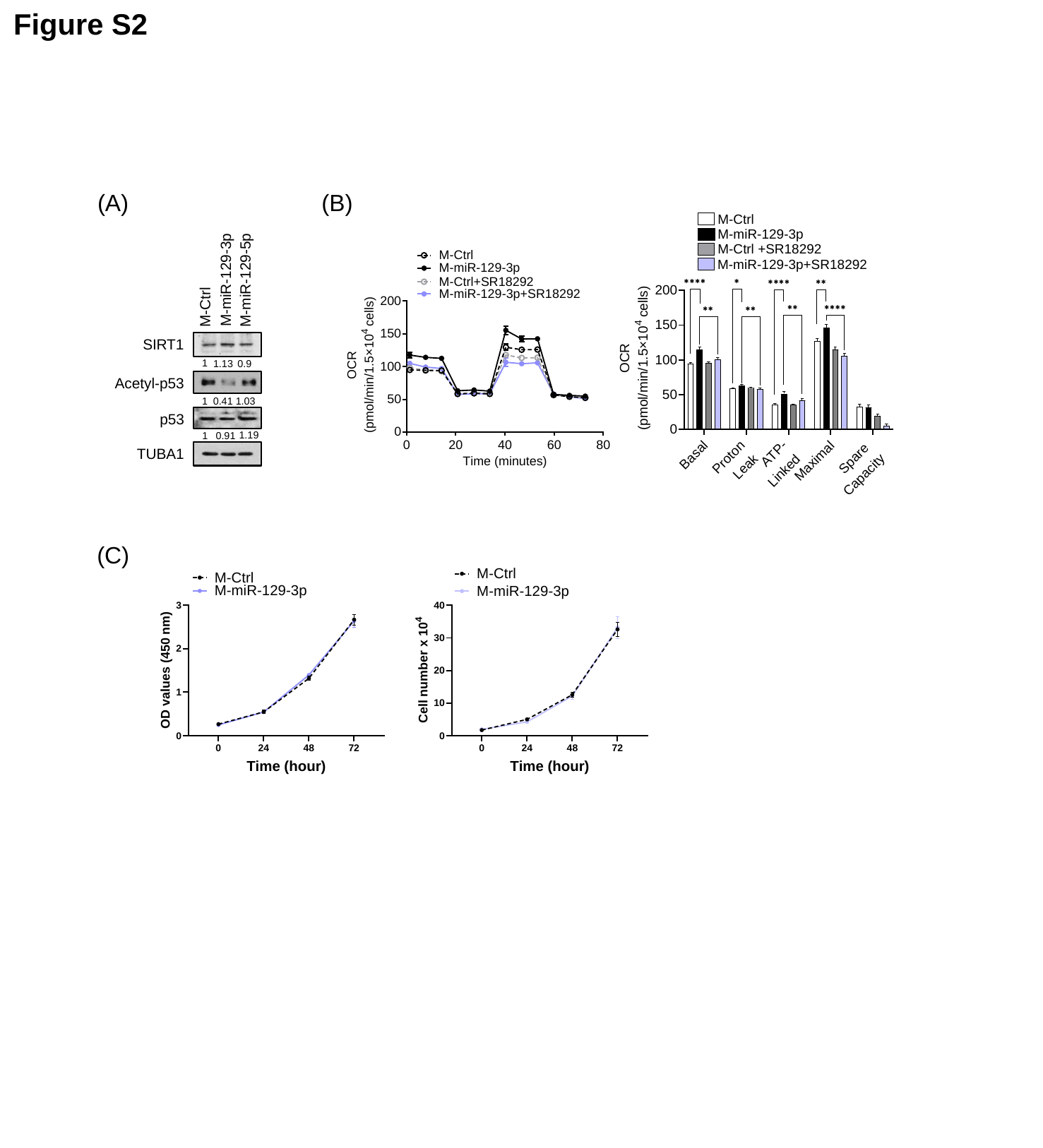

Figure S2
(A)
(B)
M-miR-129-3p
M-miR-129-5p
M-Ctrl
SIRT1
1
1.13
0.9
Acetyl-p53
1.03
1
0.41
p53
 1.19
1
0.91
TUBA1
(C)

## Slide 3
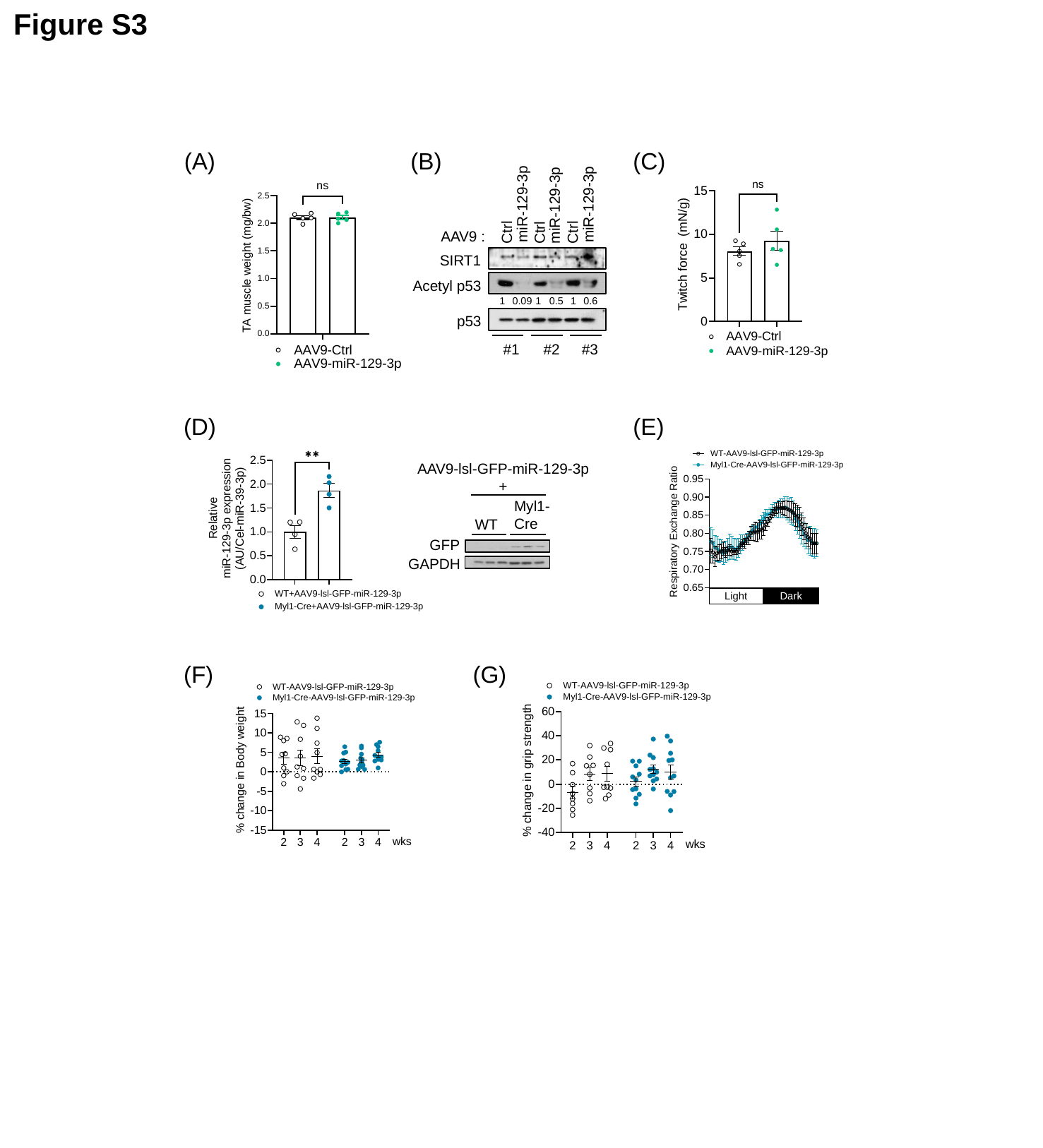

Figure S3
(A)
(B)
(C)
miR-129-3p
miR-129-3p
miR-129-3p
Ctrl
Ctrl
Ctrl
AAV9 :
SIRT1
Acetyl p53
1
0.09
1
0.5
1
0.6
p53
#1
#2
#3
(D)
(E)
AAV9-lsl-GFP-miR-129-3p
+
Myl1-
Cre
WT
GFP
GAPDH
(F)
(G)

## Slide 4
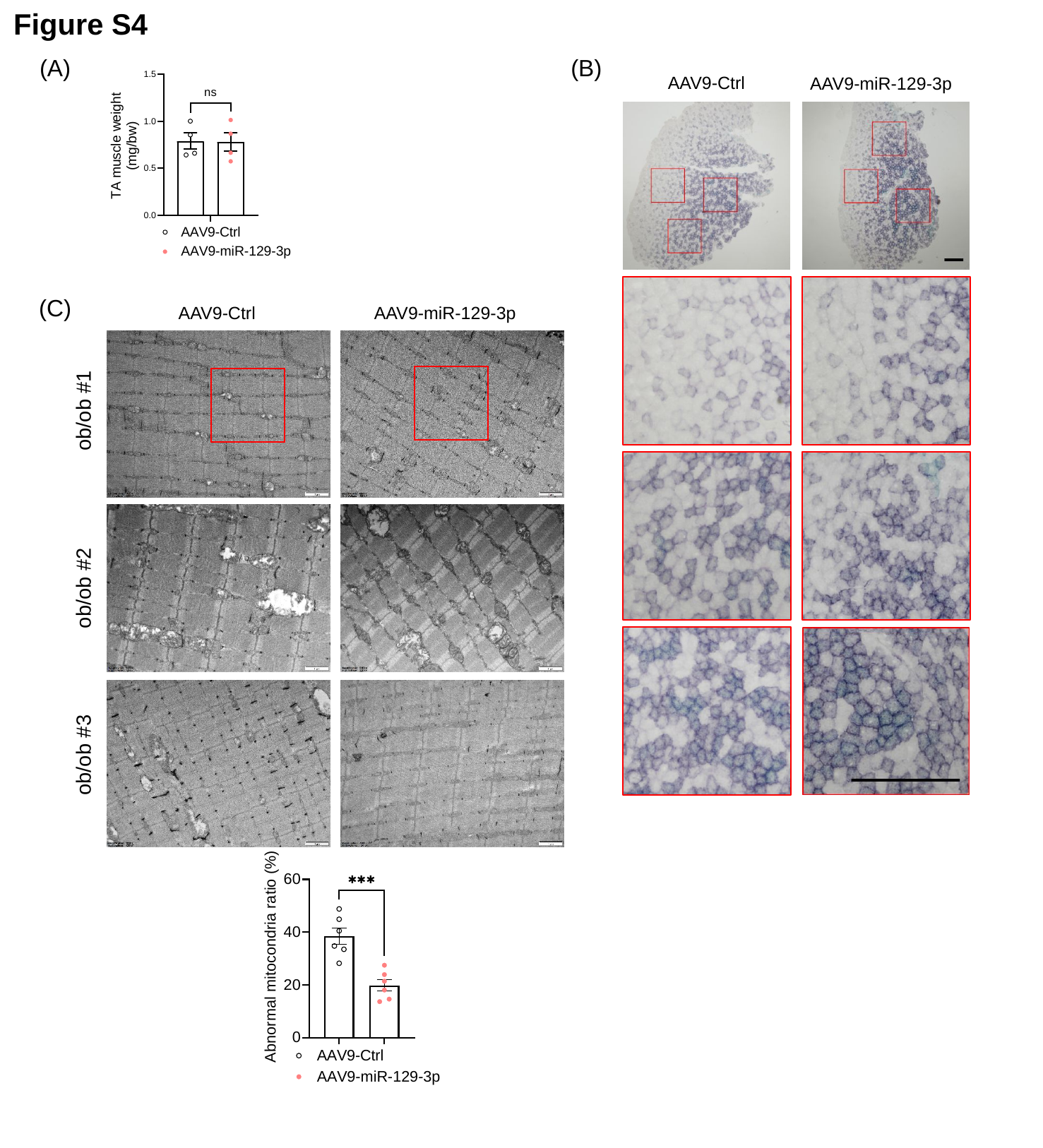

Figure S4
(A)
(B)
AAV9-Ctrl
AAV9-miR-129-3p
(C)
AAV9-Ctrl
AAV9-miR-129-3p
ob/ob #1
ob/ob #2
ob/ob #3

## Slide 5
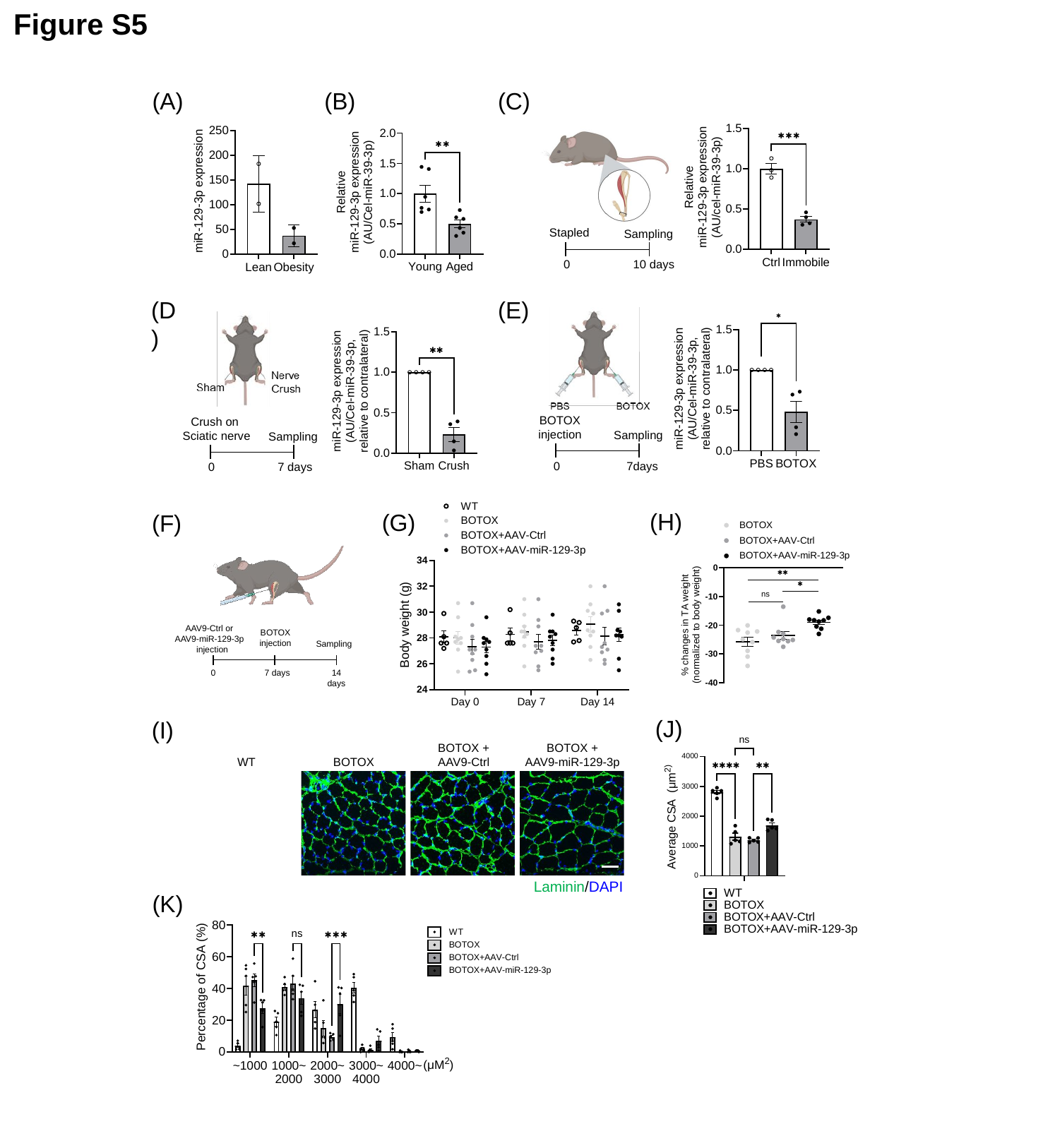

Figure S5
(A)
(B)
(C)
Stapled
Sampling
0
10 days
(D)
(E)
BOTOX
injection
Crush on
Sciatic nerve
Sampling
Sampling
0
7days
0
7 days
(H)
(G)
(F)
AAV9-Ctrl or
AAV9-miR-129-3p
BOTOX
injection
Sampling
 injection
0
7 days
14 days
(J)
(I)
BOTOX +
AAV9-Ctrl
BOTOX +
AAV9-miR-129-3p
WT
BOTOX
Laminin/DAPI
(K)

## Slide 6
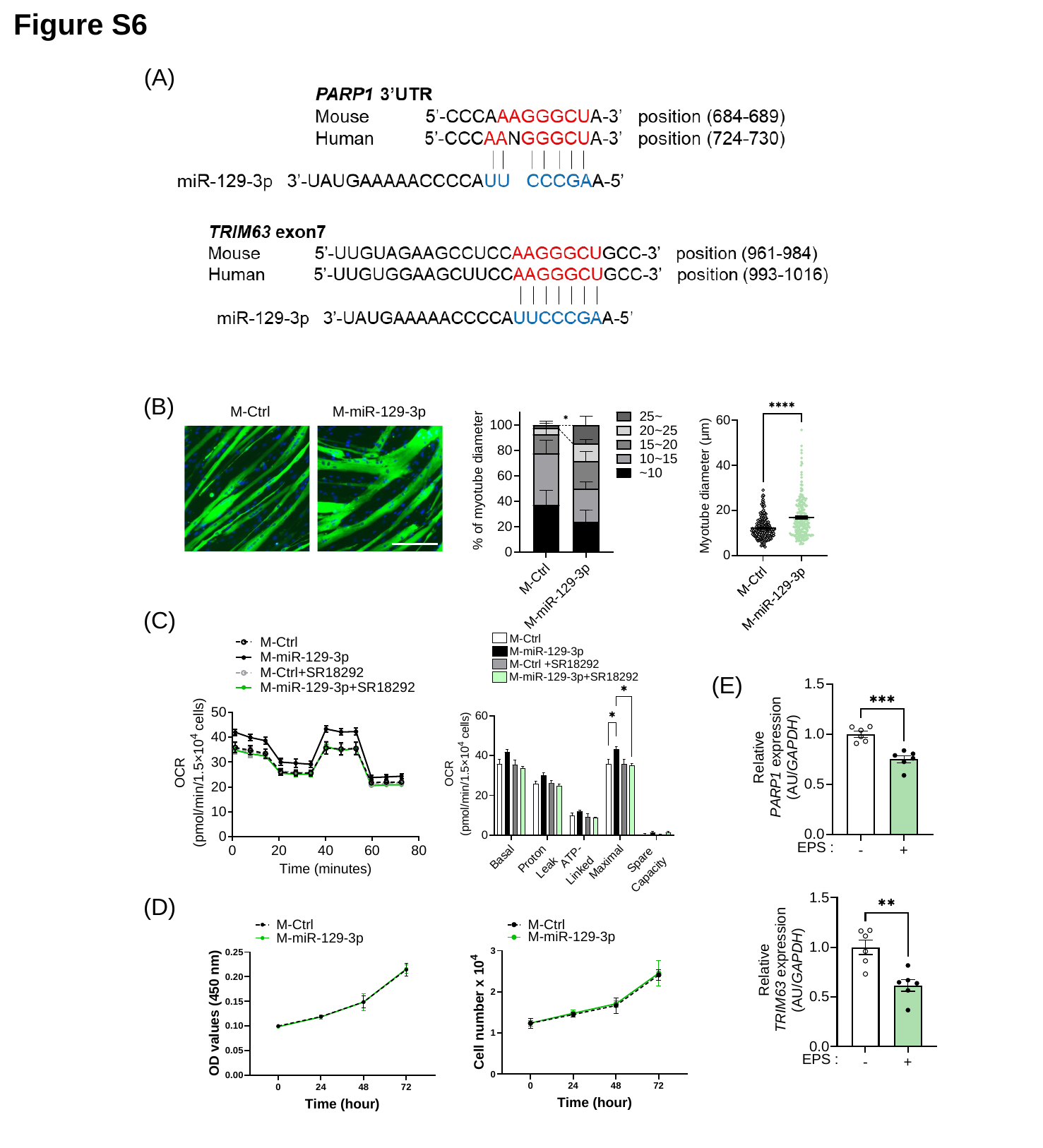

Figure S6
(A)
(B)
M-Ctrl
M-miR-129-3p
(C)
(E)
(D)
